# Crop Performance Evaluation of Chickpea and Dry Pea Breeding Lines Across Seasons and Locations Using Phenomics Data

**DOI:** 10.3389/fpls.2021.640259

**Published:** 2021-02-25

**Authors:** Chongyuan Zhang, Rebecca J. McGee, George J. Vandemark, Sindhuja Sankaran

**Affiliations:** ^1^Department of Biological System Engineering, Washington State University, Pullman, WA, United States; ^2^USDA-ARS, Grain Legume Genetics and Physiology Research, Washington State University, Pullman, WA, United States

**Keywords:** image processing, multispectral imagery, unmanned aircraft vehicle, vegetation indices, yield prediction

## Abstract

The Pacific Northwest is an important pulse production region in the United States. Currently, pulse crop (chickpea, lentil, and dry pea) breeders rely on traditional phenotyping approaches to collect performance and agronomic data to support decision making. Traditional phenotyping poses constraints on data availability (e.g., number of locations and frequency of data acquisition) and throughput. In this study, phenomics technologies were applied to evaluate the performance and agronomic traits in two pulse (chickpea and dry pea) breeding programs using data acquired over multiple seasons and locations. An unmanned aerial vehicle-based multispectral imaging system was employed to acquire image data of chickpea and dry pea advanced yield trials from three locations during 2017–2019. The images were analyzed semi-automatically with custom image processing algorithm and features were extracted, such as canopy area and summary statistics associated with vegetation indices. The study demonstrated significant correlations (*P* < 0.05) between image-based features (e.g., canopy area and sum normalized difference vegetation index) with yield (*r* up to 0.93 and 0.85 for chickpea and dry pea, respectively), days to 50% flowering (*r* up to 0.76 and 0.85, respectively), and days to physiological maturity (*r* up to 0.58 and 0.84, respectively). Using image-based features as predictors, seed yield was estimated using least absolute shrinkage and selection operator regression models, during which, coefficients of determination as high as 0.91 and 0.80 during model testing for chickpea and dry pea, respectively, were achieved. The study demonstrated the feasibility to monitor agronomic traits and predict seed yield in chickpea and dry pea breeding trials across multiple locations and seasons using phenomics tools. Phenomics technologies can assist plant breeders to evaluate the performance of breeding materials more efficiently and accelerate breeding programs.

## Introduction

Crop cultivars are consistently selected based on their productivity (quantity and/or quality), tolerance to biotic and abiotic stressors, and adaptation to local production systems and environments (Acquaah, 2009; Hatfield and Walthall, 2015). Pulse crops, including pea (*Pisum sativum* L.) and chickpea (*Cicer arietinum* L.), have been bred for their adaptation to the Palouse region in the Pacific Northwest, United States, with the overall goal of developing high-yielding and biotic and abiotic stress-resistant cultivars. The Palouse region, which includes parts of eastern Washington, northern Idaho, and northeastern Oregon, is one of the largest producers of pulse crops in the United States (USDA-NASS, 2020) and is home to several pulse breeding programs. Pulse breeders have developed and released multiple pea and chickpea cultivars with better seed yield, quality, and improved disease resistance (McGee and McPhee, 2012; McGee et al., 2012, 2013; Vandemark et al., 2014, 2015; USDA-ARS, 2018). However, plant breeders have primarily relied on traditional methods to collect phenotypic data on breeding lines. Some of the constraints of these traditional phenotyping approaches are that they are labor-intensive, time-consuming, and subjective with limited availability of data. Therefore, sensing technologies, also referred to as phenomics technologies, are needed to overcome these constraints to facilitate progress of plant breeding and provide data for a more accurate and comprehensive evaluation of breeding lines.

Plant traits evaluated by phenomics technologies in field conditions include early vigor (Kipp et al., 2014; Sankaran et al., 2015), canopy area and temperature (Patrignani and Ochsner, 2015; Bai et al., 2016), plant height (Madec et al., 2017; Wang et al., 2018), heading and flower intensity (Sadeghi-Tehran et al., 2017; Zhang et al., 2020), yield (Donohue et al., 2018; Lai et al., 2018), and phenological stages (Yang et al., 2017). Research using phenomics technologies to monitor or predict crop yield has been conducted for a wide range of crops. Different image-based plant traits, such as flowers, vegetation indices (VIs), plant height, and canopy area (Bai et al., 2016; Tattaris et al., 2016; Thorp et al., 2016; Sun et al., 2018), have been used to monitor and predict crop yield. Thorp et al. (2016) used proximal digital imaging to track *Lesquerella* flowering dynamics and reported that there was a strong correlation between flower cover percentage and seed yield (coefficient of determination or *R*^2^ ≤ 0.84). Sun et al. (2018) developed a terrestrial light detection and ranging (LiDAR)-based high-throughput phenotyping system and applied it to monitor cotton growth. Their results indicated that canopy height, projected canopy area, and plant volume (*R*^2^ ≤ 0.84, 0.88, and 0.85, respectively) at 67 and 109 days after planting were positively correlated with yield. In addition to correlating plant traits with yield, researchers have tested models to predict seed yield and biomass of wheat, canola, and corn (Fieuzal et al., 2017; Ballesteros et al., 2018; Donohue et al., 2018; Lai et al., 2018; Anderson et al., 2019). Fieuzal et al. (2017) developed two artificial neural network-based methods (a real-time approach and a diagnostic approach) to estimate corn yield using multi-temporal optical and radar satellite data. The diagnostic approach using reflectance from the red spectral region predicted yield with *R*^2^ = 0.77, while the real-time approach using reflectance from the red spectral region and one feature from radar satellite data resulted in a prediction accuracy of *R*^2^ = 0.69.

Other performance traits have also been evaluated using sensing technologies, including estimation of phenological stages, 50% flowering, senescence, and maturity (Viña et al., 2004; Yu et al., 2016; Zheng et al., 2016; Yang et al., 2017; Quirós Vargas et al., 2019; Lindsey et al., 2020). Zheng et al. (2016) monitored rice phenology in three growing seasons using a time series of spectral indices obtained using portable spectrometers. They reported that the red-edge chlorophyll index can accurately detect the dates of jointing, middle booting, and soft dough, while the normalized difference vegetation index (NDVI) can detect dates of active tillering, middle heading, and maturity. In our previous study (Quirós Vargas et al., 2019), we found that VIs, including NDVI, green red vegetation index (GRVI), and the normalized difference red-edge index (NDRE), were correlated with days to 50% flowering and physiological maturity in two winter pea experiments.

Although phenomics technologies have been tested on many crops, the evaluation of such technologies across field seasons, locations, and different crop types has been limited for pulse crops. Such efforts are essential to assess the stability and applicability of phenomics technologies to assist breeding programs. Therefore, in this study, we applied sensing technologies to evaluate dry pea and chickpea breeding lines for three growing seasons (2017–2019) for phenotyping applications. Specific objectives were to: 1) monitor yield and other agronomic traits using quadcopter unmanned aircraft vehicle (UAV) multispectral imaging data and 2) predict pulse crop yield with a multivariate regression model.

## Materials and Methods

### Experimental Locations and Plant Materials

The pulse crop (chickpea and dry pea) breeding lines in this study (2017–2019) were evaluated in multiple field locations, near Pullman, WA (46°41′39.0″N, 117°08′53.0″W), Fairfield, WA (47°19′08.0″N, 117°10′05.0″W), and Genesee, ID (46°36′40.0″N, 116°57′39.0”W), United States (Table 1). The exact locations of the experiment field sites within an area varied between years due to crop rotation protocols. Advanced yield trials of green pea (panel 01), yellow pea (panel 02), and chickpea (panel 81) breeding lines and relevant commercial check cultivars were planted using a randomized complete block design with three replicates. A seed treatment was applied prior to planting that contained the fungicides fludioxonil (0.56 g kg^–1^; Syngenta, Greensboro, NC, United States), mefenoxam (0.38 g kg^–1^; Syngenta), and thiabendazole (1.87 g kg^–1^; Syngenta), thiamethoxam (0.66 ml kg^–1^; Syngenta) for insect control, and molybdenum (0.35 g kg^–1^). Approximately 0.5 g *Mesorhizobium ciceri* inoculant (1 × 10^8^ CFU g^–1^; Novozyme, Cambridge, MA, United States) was applied to each chickpea seed packet 1 day before planting. Chickpea plots were planted at 6.1 m long and 1.5 m wide with approximately 75 cm between plots. After emergence, the plots were trimmed to approximately 4.9 m long, thus leaving alleys of approximately 1.2 m between ranges. Chickpeas were planted at a density of 43 seeds m^–2^ in a 1.5-m × 6.1 m block (≈430,000 seeds ha^–1^). The chickpea entries had either compound or simple leaf types. Typically, there were four to seven entries each year with simple leaves. The dry pea entries had either normal (cv. ‘Columbian’ only) leaves or were semi-leafless. Data analysis was conducted without separating the leaf types for each crop, and preliminary analysis indicated that the ranges of the vegetation indices were similar.

**TABLE 1:** Summary of the pulse crops’ breeding trials and data acquisition using sensing.

Year	Location	Sensing altitude^*a*^	Crop	Panel^*b*^	Number of lines/cultivars	Sowing date	Data acquisition dates	Growing degree days^*c*^
2017^*d*^	Pullman	45 m	Chickpea	1781	24	5/11	6/26, 7/07, 7/21, 7/28	536, 723, 959, 1087
			Dry pea	1701, 1702	40, 21	5/11	6/26, 7/07, 7/21	536, 723, 959
	Fairfield	45 m	Dry pea	1701, 1702	40, 21	5/11	7/24^*e*^	1077
2018	Pullman	25 m	Chickpea	1881	21	5/05	6/08, 6/22, 7/03, 7/19, 7/27	423, 582, 721, 991, 1124
			Dry pea	1801, 1802	32, 23	5/05	6/08, 6/22, 7/03, 7/19, 7/27	423, 582, 721, 991, 1124
	Genesee	25 m	Chickpea	1881	21	5/08	6/08, 6/26, 7/09, 7/23, 8/06	372, 576, 752, 997, 1274
			Dry pea	1801	32	5/08	6/08, 6/26, 7/09, 7/23, 8/06	372, 576, 752, 997, 1274
	Fairfield	25 m	Chickpea	1881	21	5/21	6/12, 6/29, 7/12, 7/25	282, 515, 716, 966
			Dry pea	1801, 1802	32, 23	5/21	6/12, 6/29, 7/12, 7/25, 8/07	282, 515, 716, 966, 1274
2019	Pullman	30 m	Chickpea	1981	24	5/04	6/05, 6/17, 7/05, 7/16	387, 549, 772, 939
			Dry pea	1901, 1902	29, 23	5/04	6/05, 6/17, 7/05, 7/16	387, 549, 772, 939
	Genesee	25 m	Chickpea	1981	24	5/03	6/05, 6/18, 7/05, 7/16	384, 559, 756, 923
			Dry pea	1901	29	5/03	6/05, 6/18, 7/05, 7/16	384, 559, 756, 923
	Fairfield	30 m	Chickpea	1981	24	5/06	6/10, 6/28, 7/12, 7/23	425, 679, 902, 1080
			Dry pea	1901, 1902	29, 23	5/06	6/10, 6/28, 7/12	425, 679, 902

*^*a*^Sensing altitudes (25, 30, and 45 m) as above ground level used in this study resulting in 1.7, 2.0, and 3.1 cm per pixel of ground sampling distance.*

*^*b*^Panels ending in “01” are advanced green pea, “02” are advanced yellow pea, and “81” are advanced chickpea.*

*^*c*^Growing degree days: accumulated degree days that are used to estimate temperature-based growing season; degree day = mean temperature - base temperature (base temperature = 3°C in this study) (Bourgeois et al., 2000; Miller et al., 2018).*

*^*d*^No data were available due to failed trials in Genesee, ID.*

*^*e*^Data from other time points were not useful (e.g., senesced plants).*

### Data Acquisition

**FIGURE 1:**
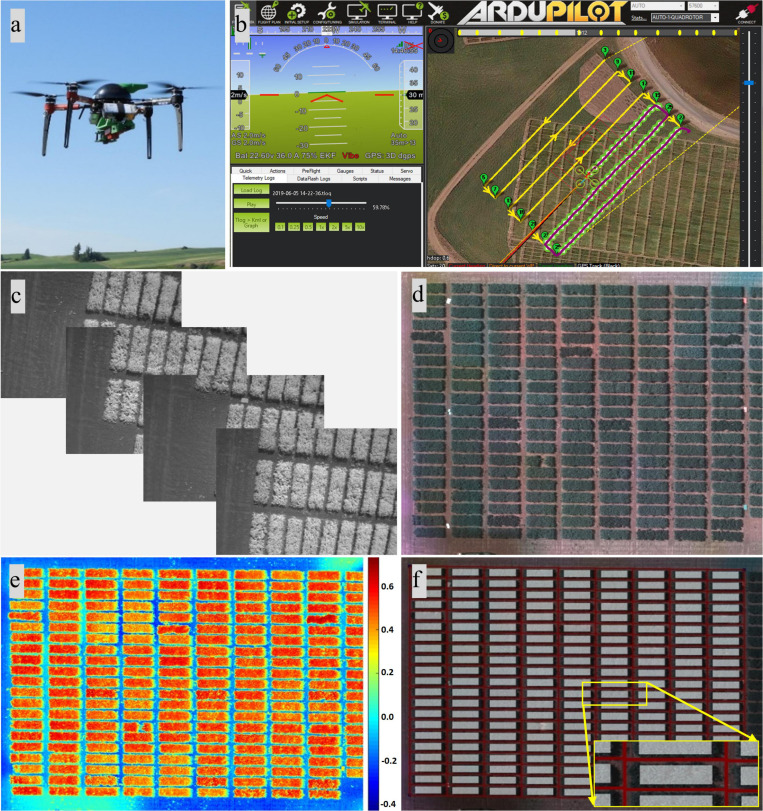
Unmanned aircraft vehicle (UAV)-based data acquisition and image processing. **(a)** UAV and camera. **(b)** Mission Planner showing data acquisition underway. **(c)** Individual images. **(d)** Orthomosaic composite images consisting of red, green, and blue bands. **(e)** Heat map of the soil-adjusted vegetation index. **(f)** Resulting image with the plot separated and the region of interest highlighted.

A quadcopter UAV (AgBot, ATI Inc., Oregon City, OR, United States) and a five-band multispectral camera (RedEdge, MicaSense Inc., Seattle, WA, United States) were deployed to acquire image data during the 3-year study (Figure 1a). The multispectral camera mounted on the quadcopter acquired images (12-bit image) with a resolution of 1.2 MP in the spectrum of red (R, 668 ± 5 nm, central band and band width), green (G, 560 ± 10 nm), blue (B, 475 ± 10 nm), near-infrared (NIR, 840 ± 20 nm), and red edge (RE, 717 ± 5 nm). Mission Planner, an open-source ground control station software (ArduPilot Development Team and Community), was used to plan and monitor missions of aerial data acquisition (Figure 1b). The UAV was programmed with Mission Planner to fly at a speed of 2–3 m/s and at 25, 30, or 45 m above ground level (AGL), resulting in a ground sampling distance (GSD) of 1.7, 2.0, or 3.1 cm per pixel, respectively, and to acquire images with 80% horizontal and 70% vertical overlaps. A reflectance panel, either a MicaSense reflectance panel (RedEdge, MicaSense Inc.) in 2017 or a Spectralon reflectance panel (99% reflectance; Spectralon, SRS-99-120, Labsphere Inc., North Sutton, NH, United States) in 2018 and 2019, was placed in the field during image acquisition and used for radiometric calibration during image processing. Data were acquired between 10:00 a.m. and 3:00 p.m. local time, and three to five time points of data acquisition were achieved for each season (Table 1). The time points for data acquisition were selected to acquire data representing key growth stages, such as early growth, flowering, and seed/pod development stages, and based on suitable weather conditions for UAV flights (e.g., clear sky and low wind). Seed yield data from the dry pea and chickpea trials were collected from each location, while other agronomic and phenological traits were collected only at Pullman each year. These traits include days to 50% flowering, days to physiological maturity, pod height, pod height at maturity, overall vine length, canopy height at maturity, node of first flower, and number of reproductive nodes.

### Image Processing and Feature Extraction

**FIGURE 2:**
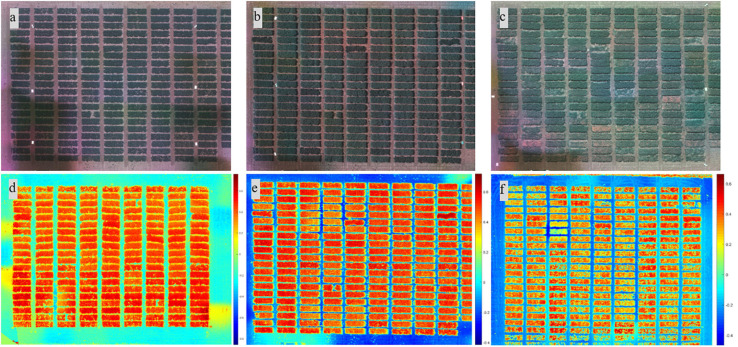
Composite RGB images from different growth stages (2018) and heat map of the soil-adjusted vegetation index from different locations. Images from the **(a)** early, **(b)** flowering, and **(c)** pod/seed development stages. Heat map of the soil-adjusted vegetation index from **(d)** Genesee, **(e)** Pullman, and **(f)** Fairfield.

Images from the multispectral camera (Figure 1c) were first processed in Pix4Dmapper (Pix4D Inc., San Francisco, CA, United States) to generate orthomosaic images covering each experimental site. The template used in Pix4Dmapper was based on Ag Multispectral, where the calibration method of “Alternative” was selected in the initial processing. In this type of calibration, the images are optimized for aerial nadir images with accurate geolocation information, low texture content, and relatively flat terrain. Orthomosaic images were imported into custom semi-automated image processing algorithms developed in MATLAB (2018b; MathWorks Inc., Natick, MA, United States) for further processing. The image processing algorithms prompted the user to input a degree to rotate the image, which is prepared for plot segmentation later, and to identify the reflectance panel for radiometric calibration. Following that, composite RGB image and several vegetation index maps were generated using different combinations of orthomosaic images (Figures 1d,e,2). A composite RGB image was generated for quality inspection. The vegetation indices calculated included normalized difference vegetation index (NDVI), green NDVI (GNDVI), soil-adjusted vegetation index (SAVI), normalized difference red-edge index (NDRE), and triangular vegetation index (TVI) (Harris Geospatial Solutions, 2020). For each dataset (each crop and each data acquisition period), a master mask that separated the canopy from the background, such as soil and flowers (for dry pea only), was generated by setting a threshold on the SAVI index map prior to feature extraction. Threshold data varied between datasets based on canopy vigor and illumination conditions at the time of data acquisition, and the value was selected for each dataset based on visual assessment.

In dry pea, flowers that are white have a different reflectance than stipules and tendrils and were excluded from canopy feature extraction. A similar procedure was not applied to chickpea as chickpea flowers could not be detected in five-band multispectral images at the given resolution due to the small flower size (Zhang et al., 2020). The master mask was overlaid on the composite RGB image for quality inspection and optimization of the threshold for generating a canopy mask. The developed algorithm prompted the user to identify the four corners of the field and automatically separated individual plots with information of the identified corners (Figure 1f). Masks for individual plots were then shrunk at the four edges to prevent edge effects, forming regions of interest for each plot that were highlighted with white, as shown in Figure 1f. The top and bottom edges of the mask for an individual plot were reduced by 11 (2017) or 20 (2018 and 2019) pixels, while the right and left edges were reduced by 28 (2017) or 50 (2018 and 2019) pixels. More details about the algorithm can be found in Zhang et al. (2019). Image-based features were extracted from regions of interest in each plot, including canopy area (in pixels), and the mean and sum statistic of NDVI, GNDVI, SAVI, NDRE, and TVI plot images. Here, the mean of NDVI, for example, is the average of the NDVI values of the canopy pixels identified by the mask for an individual plot, while the sum of NDVI is the sum of the NDVI values of canopy pixels. At the end of image processing, the algorithms exported the features as Excel files for further analysis. The procedures of image processing for dry pea and chickpea were similar with minor modifications, such as the threshold used to create the master mask.

### Data Analysis

Image-based features from the UAV data were analyzed using Pearson’s correlation in SAS, University Edition (SAS Institute, Cary, NC, United States). The features were correlated with yield for all locations and with other traits for the Pullman trial only due to availability of data. Plot-by-plot and cultivar-by-cultivar (by averaging the replicates at each field site) correlation analyses were also conducted. Noisy data (e.g., cloud-covered plots) were eliminated prior to analysis.

Yield prediction models were developed using image-based features as predictors to estimate yields in the chickpea (panel 81) and green pea (panel 01) trials. Due to the availability of data across the three locations, only green pea breeding lines were utilized for yield prediction. Yields were predicted using data from each year and each location (2017: Pullman; 2018 and 2019: Pullman, Genesee, and Fairfield) and the combined data for each year (2018 and 2019). The identity of the breeding lines varied from year to year as lines were discarded or added to the panels, and therefore data were only combined within a year. In 2017, no chickpea data were available from Genesee and Fairfield, and only one set of pea data was available from Fairfield.

**FIGURE 3:**
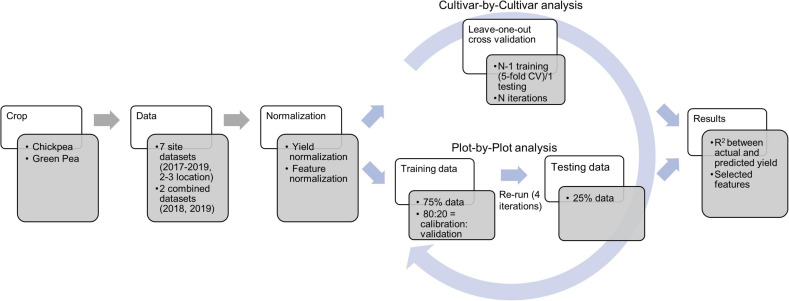
Workflow of the data analysis during yield prediction using the least absolute shrinkage and selection operator (LASSO) model. *CV* and *N* refer to cross-validation and number of lines/cultivars, respectively.

Datasets acquired at flowering, and pod and seed development stages were utilized for yield model development. The data (yield and image features) were normalized using the following formula prior to the model development (Figure 3).

(1) $$x_{\text{n}}=\frac{\left(x_{r}-m\right)}{\sigma }$$

where *x*_*n*_, *x*_*r*_, *m*, and *σ* stand for normalized data, raw data, mean of a feature, and standard deviation of a feature, respectively. Normalization was conducted for each year and each location as well as for the combined data within each year. Yield prediction was conducted using both plot-by-plot data and cultivar-by-cultivar data (average of three replicate data across each field site). Least absolute shrinkage and selection operator (LASSO) regression programmed in MATLAB was used in this study for yield prediction. The parameters used in LASSO included: alpha (weight of lasso *versus* ridge optimization) = 1, MCReps (repetitions of cross-validation) = 3, cross-validation = five-fold, and predictor selection method (for cross-validation) = IndexMinMSE. More details about LASSO in MATLAB can be found at the MathWorks website^1^.

For the plot-by-plot analysis, the dataset was divided into the training and the testing datasets with a ratio of 3:1. The training data were further resampled five times (80% of training data to calibrate and 20% of training data to validate) to optimize the models. Finally, the model was evaluated using the test dataset and the process was assessed four times (four iterations) to eliminate effects of randomization. For the cultivar-by-cultivar analysis, due to the limited sample size, the leave-one-out approach for model development and evaluation was utilized (Sammut and Webb, 2010). During model development, a five-fold cross-validation was used, followed by testing the model for as many times as the instances (29–40 lines/cultivars depending on the dataset). The prediction performance was reported in terms of *R*^2^ during the train and test processes and selected image-based features.

## Results

### Relationship Between Image Features and Yield

**FIGURE 4:**
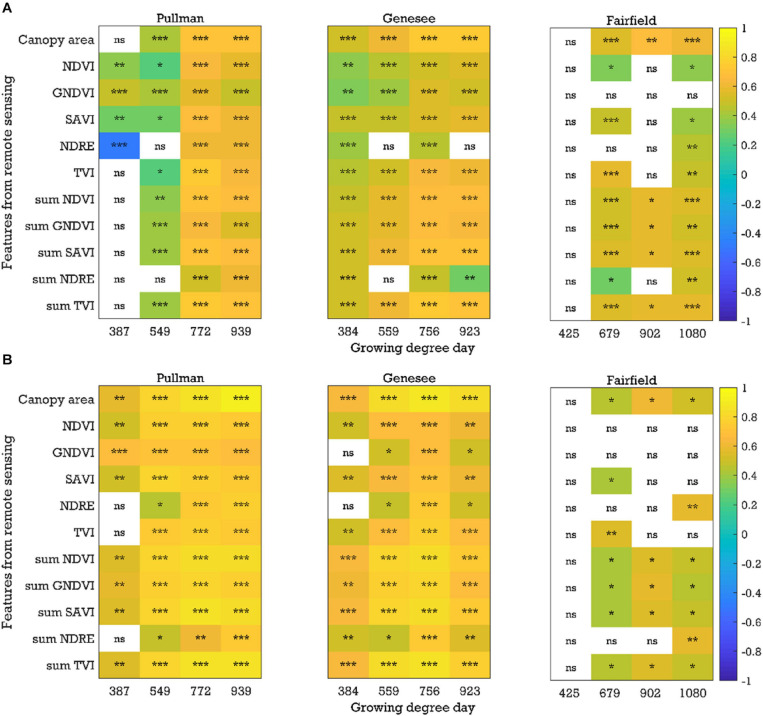
Correlation coefficients between the image-based features and yield for the chickpea yield trial in 2019: **(A)** plot-by-plot analysis and **(B)** cultivar-by-cultivar analysis. *NDVI*, normalized difference vegetation index; *GNDVI*, green *NDVI*; *SAVI*, soil-adjusted vegetation index; *NDRE*, normalized difference red-edge index; *TVI*, triangular vegetation index; *NDVI*, for example, is the average of the NDVI values of canopy pixels, while *sum NDVI* is the sum of the NDVI values of canopy pixels; *ns*, nonsignificant at the 0.05 probability level. Significant probability levels: *0.05, **0.01, and ***0.001.

In general, there were significant and positive correlations (*P* < 0.05, *r* up to 0.74) between image-based features (e.g., canopy area, SAVI, and sum NDVI) and yield with the plot-by-plot chickpea data acquired at the early growth, flowering, and pod/seed development stages across field seasons (2017–2019) and locations (Figure 4A and Supplementary Figure 1). Chickpea and dry pea flowered between 721 and 772 growing degree days. Plants were considered in the early growth stages before flowering and in the pod/seed development stage between the flowering stage and physiological maturity. Only a few common image features (e.g., NDVI and SAVI) extracted from the data acquired at the early growth stages were significantly correlated with yield across seasons and experimental locations, while more common image features (e.g., canopy area, NDVI, SAVI, sum of NDVI, GNDVI, and SAVI) extracted from the data acquired at the flowering and pod/seed development stages were correlated with yield. A similar trend was found when analyzing the chickpea data using the cultivar-by-cultivar method (*r* up to 0.93). During the cultivar-by-cultivar data analysis, fewer features were significantly correlated with yield (Figure 4B and Supplementary Figure 2), which could be due to the smaller dataset compared to the plot-by-plot analysis method. In most cases, the correlations between the image features and yield were greater from data acquired at the flowering and pod/seed development stages in comparison to the early growth stages.

**FIGURE 5:**
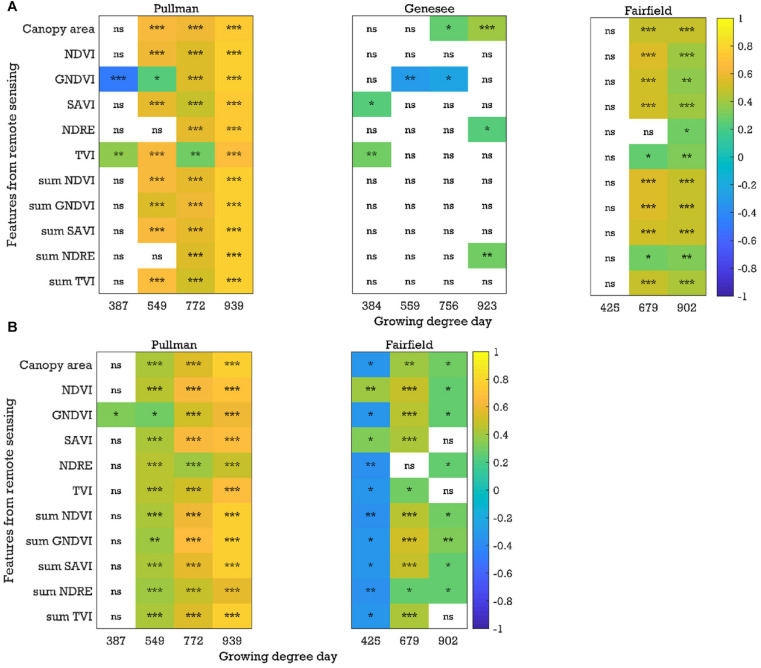
Correlation coefficients between the image-based features and yield for the **(A)** green pea and **(B)** yellow pea trials in 2019 (plot-by-plot analysis). *NDVI*, normalized difference vegetation index; *GNDVI*, green NDVI; *SAVI*, soil-adjusted vegetation index; *NDRE*, normalized difference red-edge index; *TVI*, triangular vegetation index; *NDVI*, for example, is the average of the NDVI values of canopy pixels, while *sum NDVI* is the sum of the NDVI values of canopy pixels; *ns*, nonsignificant at the 0.05 probability level. Significant probability levels: *0.05, **0.01, and ***0.001.

Similar to chickpea, in the green pea breeding lines, significant positive correlations (*P* < 0.05, *r* up to 0.83) between the image-based features (e.g., canopy area and sum NDVI) extracted from the plot-by-plot data acquired at the early growth, flowering, and pod/seed development stages and yield were observed across field seasons and experimental locations in most cases (Figure 5A and Supplementary Figure 3). When analyzing the cultivar-by-cultivar data, typically fewer image features within a time point were significantly correlated with yield, although the *r* values were up to 0.80 (Supplementary Figure 4). It was interesting to note that four of eight trials (field seasons × experimental locations) showed significant negative correlations between yield and image features from the data acquired at the early growth/pre-flowering stages using both analysis methods (plot-by-plot or cultivar-by-cultivar). The potential reason for the negative relationships between the image-based features (e.g., NDVI data) at the early growth stages and yield is unclear and requires further investigation. We have observed that pea cultivars that flower early typically have better early seedling vigor; however, they also have lower seed yields, presumably because the plants do not have an extended vegetative period during which they can produce as much photosynthate (and hence seeds) as later flowering cultivars. In peas, the timing of flowering is dictated by photoperiod response rather than by biomass accumulation. In general, phenotyping the pea trials was more challenging than for chickpea, which may be due to the presence of tendrils in the cultivars. The spectral reflectance of tendrils may be different from that of stipules. Similar patterns of significant correlations (*P* < 0.05, *r* up to 0.85) with data acquired at the early growth, flowering, and pod/seed development stages were found in the yellow pea yield trials (Figure 5B and Supplementary Figures 5,6). Significant negative correlations at the early stage were rare in the yellow pea yield trials, unlike the green pea yield trials.

### Relationship Between Image Features and Other Data Types

**FIGURE 6:**
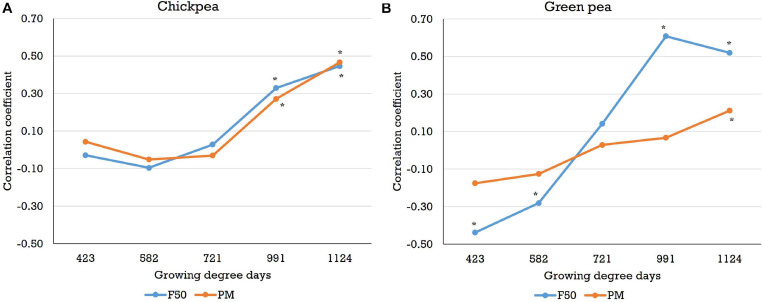
Correlations between the sum normalized difference vegetation index and days to 50% flowering (*F50*) or days to physiological maturity (PM) for **(A)** chickpea and **(B)** green pea in the 2018 field season (plot-by-plot analysis). *Sum NDVI* is the sum of the NDVI values of canopy pixels. Correlations that are significant at the 0.05 probability level are indicated by an *asterisk*.

Correlations between the image-based features (e.g., NDVI, SAVI, and sum SAVI) and other traits (e.g., days to 50% flowering, days to physiological maturity, plant height, pod length, etc.) acquired from the Pullman trials were analyzed across three field seasons. For the chickpea yield trials, significant (*P* < 0.05) and positive correlations between the image-based features and days to 50% flowering or days to physiological maturity (*r* up to 0.76 and 0.58, respectively) were found after the flowering stage (Figure 6 and Supplementary Figures 7,8). Most of the negative correlations observed between the image-based features and days to 50% flowering or days to physiological maturity at the early growth stages were not significant. On the other hand, correlations between the image-based features and the remaining traits were not consistent across the three field seasons (data not presented).

With regard to the green pea yield trials, significant and negative correlations (*P* < 0.05, *r* > −0.54) between features (e.g., NDVI, SAVI, and sum SAVI) and days to 50% flowering were observed at the early stages (Figure 6 and Supplementary Figure 9) across 3 years for both analysis methods in most cases. Negative correlations between features and days to physiological maturity were also observed in the early growth stages, although most correlations were not significant (Figure 6 and Supplementary Figure 10). Early plant vigor (higher vegetation index data) may be associated with early flowering and maturity (early flowering/maturity dates), which would result in negative correlations. As with chickpea, most image-based features acquired after flowering were significantly and positively correlated with days to 50% flowering or days to physiological maturity (*r* up to 0.75 and 0.72, respectively), especially when the images were acquired close to physiological maturity. No consistent trends in the correlations between the image-based features and the other traits were found. In the yellow pea yield trials, negative correlations between features and days to 50% flowering or days to physiological maturity were also observed in the early growth stages. However, significant positive correlations (*r* up to 0.85 and 0.84, respectively) between these two traits and most of the image-based features were found in the datasets acquired after flowering (Supplementary Figures 11,12). In the yellow pea yield trials, there were some image features (e.g., NDVI, SAVI, and sum SAVI) that were significantly correlated with pod height index (negative) and pod height (positive) across the three field seasons, especially in the pod/seed development stage (data not presented).

### Yield Prediction Using LASSO Regression

**FIGURE 7:**
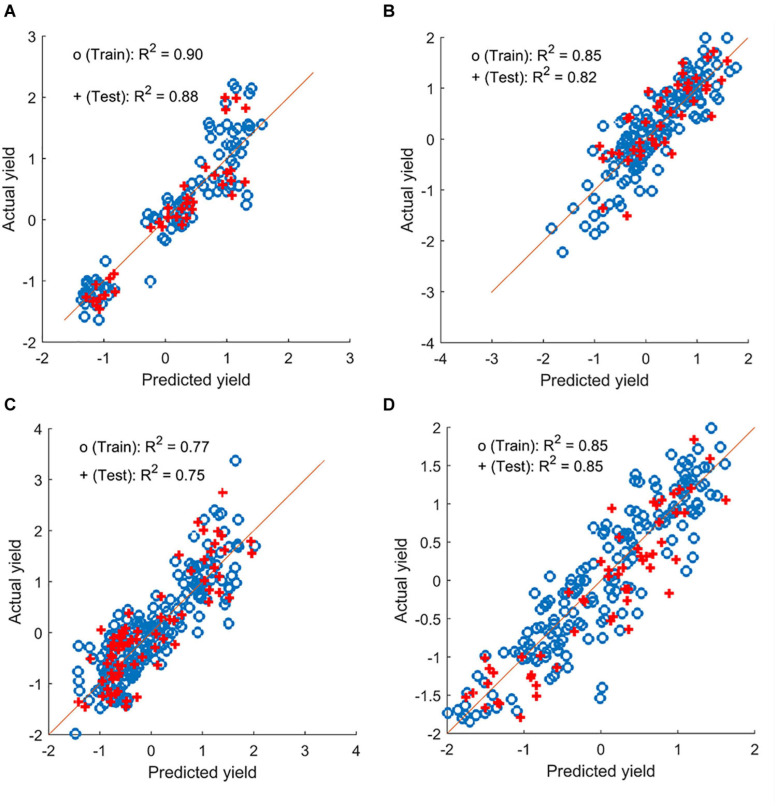
Yield prediction results from a sample iteration using the combined data for chickpea and pea (plot-by-plot analysis). **(A,B)** Chickpea datasets from 2018 and 2019. **(C,D)** Green pea datasets from 2018 and 2019, respectively.

Chickpea yield can be predicted using multiple image-based features, as summarized in Table 2. The prediction accuracy varied across field seasons and locations, regardless of the analysis method, with testing accuracy (for individual locations) of up to 0.84. When the data within a year were combined, the prediction accuracy increased in 2018 and 2019 (testing accuracy of up to 0.91; Figure 7). Regardless of whether the data were separated for individual locations or combined within a year, the features selected by LASSO as predictors varied from one to seven. Only features that were selected at least 75% of the time during multiple runs of model development were considered. Among these features, canopy area and NDRE or sum of NDRE were usually selected as predictors. Features derived from the data collected at the flowering and pod/seed development stages were both selected during model development, indicating similar importance of these two stages.

**TABLE 2:** Yield prediction results of the models for chickpea crop.

Year	Location	Plot-by-plot method			Cultivar-by-cultivar method		
		Train R^2^	Test R^2^	Number of features^*a*^	Train R^2^	Test R^2^	Number of features^*a*^
2017	Pullman	0.55	0.32	4	0.61	0.11	2
2018	Pullman	0.63	0.53	3	0.77	0.42	6
	Genesee	0.45	0.33	3	0.52	0.31	3
	Fairfield	NA	NA	NA	NA	NA	NA
	Combined	0.90	0.89	5	0.93	0.91	4
2019	Pullman	0.74	0.57	3	0.86	0.84	1
	Genesee	0.67	0.51	6	0.83	0.75	2
	Fairfield	0.79	0.70	2	NA	NA	NA
	Combined	0.84	0.82	7	0.86	0.76	5

*^*a*^Only features with ≥ 75% selection occurrence during multiple iterations/runs during model development were considered.*

*NA indicates failure to develop yield prediction model.*

Yield in the green pea yield trials can also be estimated by multiple image-based features using data from individual locations and combined within a year (Supplementary Table 1). Prediction (testing) accuracy reached up to 0.72 and 0.80 for the data from individual locations and the combined datasets within a year (e.g., Figure 7), respectively. Similar to chickpea, the features derived from the images acquired at flowering and at pod/seed development were both selected as predictors. However, more features (3–20) were used in the model development for this panel, and more models performed poorly when analyzing the cultivar-by-cultivar data. These may be related to the complicated pea canopy architecture that comprised stipules and tendrils. Among these features, canopy area, NDRE, and TVI were selected more often as predictors than other features.

## Discussion

The study demonstrates that image-based features including canopy area, NDVI, SAVI, and sum NDVI derived from UAV data can be used to monitor performance traits such as yield, days to 50% flowering, or days to physiological maturity across experimental locations and field seasons in two pulse crops, chickpea and pea. Phenomics technologies, especially UAV-based multispectral imaging systems, can be used to acquire data in a standard, rapid, and high-throughput manner, providing plant breeders with information for more informative decision making. Data on other agronomic and phenological traits, such as days to 50% flowering, days to physiological maturity, and plant height, are limited to one location or acquired at a low data acquisition frequency, often due to resource limitations, especially given the number of and the distance to the trial sites. However, using UAV integrated with multispectral camera, image data can be acquired within 30 min per trial (including setting up the UAV system), and multiple locations and crops can be imaged in a single day (depending on the distance to the trial sites). The efficiency of phenomics technologies can improve the availability of such data across multiple locations, which allows plant breeders to study the interaction between genotypes and the environment on the morphological or phenological traits.

Besides ensuring data availability, phenomics technologies can monitor a wide range of traits in pulse and other crops, including plant height and lodging (Watanabe et al., 2017; Quirós Vargas et al., 2019), disease (Marzougui et al., 2019; Zhang et al., 2019), flower intensity (Yahata et al., 2017; Zhang et al., 2020), and other traits, as discussed in this study. In addition, new traits can be derived from high temporal resolution data, such as crop growth and development curves based on canopy area, vigor, and plant height (Chang et al., 2017; Malambo et al., 2018), allowing plant breeders to assess development of each cultivar quantitatively and intensively. Current and previous studies demonstrated that seed yield or biomass of pulse or other crops can be predicted with image-based features (Fieuzal et al., 2017; Yue et al., 2017; Anderson et al., 2019; Li et al., 2019; Sankaran et al., 2019; Moghimi et al., 2020). Different machine learning models, such as LASSO, SVM, and deep neural networks, have been tested for yield prediction. For example, Moghimi et al. (2020) applied deep neural networks along with aerial hyperspectral images to predict wheat yield, which demonstrated coefficients of determination of 0.79 and 0.41 at the subplot and plot scales, respectively. Yue et al. (2017) selected the 10 most important variables among 172 variables, which were derived from multispectral and RGB images, with random forest and LASSO and used the selected variables to predict wheat yield through support vector machine (SVM) and ridge regression. Their study showed that SVM with random forest-selected variables (*r* = 0.36–0.77) and ridge regression with LASSO-selected variables (*r* = 0.40–0.73) slightly outperformed those with all variables (*r* = 0.25–0.72 and 0.22–0.73, respectively). In this regard, we found similar or better results in the current study, especially with the combined datasets. Such performance monitoring technologies can be applied in agricultural production as well as plant breeding to plan agronomic operations and save labor costs and time.

Although promising results were found in this study, some observations need further investigation. In some high-yielding trials (e.g., 2018 Pullman trial), low correlation coefficients and prediction accuracy with image-based features were observed compared to other seasons and locations. Similar observations were found in dry bean studies (Sankaran et al., 2018, 2019). One possible explanation that Sankaran et al. (2019) proposed may be that low canopy vigor resulted in great differences in the vegetation index values, which led to stronger correlations between ground truth and the vegetation index values.

Further research is also necessary to build more robust yield prediction models and confirm the potential yield predictors. Although it is possible to predict the seed yield of chickpea and dry pea, the image-based features selected in the prediction models varied across locations, years, and analysis methods. Yield prediction should be more consistent across locations and seasons with universal or common features. In general, the performance of machine learning models improves with increased data quantity and quality, which may be exploited in future study. Additional features can also be considered when building such robust prediction models, such as modified chlorophyll absorption ratio index, photochemical reflectance index, normalized difference infrared index (Harris Geospatial Solutions, 2020), plant height (Bendig et al., 2015; Rueda-Ayala et al., 2019), and canopy temperature (Sankaran et al., 2019; Zhang et al., 2019).

One of the challenges of phenotyping dry pea crop is its unique canopy architecture. The canopies of many crops, such as wheat, rice, corn, and soybean, consist of only leaves for a majority of the growing season with flowers among the canopy for a short period of time. In contrast, the pea canopy is made up of stipules and leaflets and/or tendrils for the majority of the growing season, and the tendrils may have different spectral reflectance characteristics from stipules or leaflets, which could have contributed to the lower performance of dry pea than chickpea in this study. Further study is required to identify the spectral reflectance characteristics of tendrils and stipules in pea and its relationship to crop performance.

## Conclusion

This study was conducted to evaluate phenomics technologies for monitoring performance traits (e.g., seed yield, days to 50% flowering, and days to physiological maturity) and predict the seed yield of chickpea and pea in three growing seasons and three environments (or locations). Significant correlations (*P* < 0.05) between the image features derived from multispectral UAV-based imagery and the yields of chickpea (*r* < 0.93) and pea (*r* < 0.85) were observed at the early growth, flowering, and pod/seed development stages, with a few exceptions. During seed yield prediction with the combined features dataset using LASSO regression, *R*^2^ values up to 0.91 and 0.80 (model testing) were achieved for chickpea and pea, respectively. The image-based features were identified by the LASSO regression models as the yield predictors for chickpea (one to seven features) and pea (3–20 features). The results indicated that phenomics technologies can be employed to collect data and evaluate pulse crop performance in multiple field seasons and environments and save labor and time for plant breeders. With further refinement (e.g., a software platform for data management and image analysis), phenomics technologies can be used to assist plant breeders in evaluating the performance of breeding materials and accelerate the development of new cultivars of pulse and other crops.

## Data Availability Statement

The raw data supporting the conclusions of this article will be made available by the authors, without undue reservation.

## Author Contributions

CZ and SS contributed to conceptualization, methodology, formal analysis, software development, visualization, and writing of the original draft. SS, RM, and GV helped with funding acquisition and resources. CZ, SS, RM, and GV did the data curation and investigation. All authors writing, reviewed, and edited the manuscript.

## Conflict of Interest

The authors declare that the research was conducted in the absence of any commercial or financial relationships that could be construed as a potential conflict of interest.

## Footnotes

1 https://www.mathworks.com/help/stats/lasso.html
