# Supplementary material for: Crop Performance Evaluation of Chickpea and Dry Pea Breeding Lines Across Seasons and Locations Using Phenomics Data
Source: Front Plant Sci. 2021 Feb 25;12:640259. doi: 10.3389/fpls.2021.640259 (PMC7947363; doi:10.3389/fpls.2021.640259)
Supplement: Supplementary file 1 [file Data_Sheet_1.docx]

Supplementary Material

# Supplementary Figures and Tables

## Supplementary Tables

Supplementary Table S1. Yield prediction results of the models for green pea crop.

| **Year** | **Location** | **Plot-by-plot method** | | |  | **Cultivar-by-cultivar method** | | |
| --- | --- | --- | --- | --- | --- | --- | --- | --- |
|  |  | **Train R^2^** | **Test R^2^** | **Number of**  **features ^a^** |  | **Train R^2^** | **Test R^2^** | **Number of**  **features ^a^** |
| 2017 | Pullman | 0.84 | 0.72 | 4 |  | 0.34 | 0.18 | 3 |
| 2018 | Pullman | NA | NA | NA |  | NA | NA | NA |
|  | Genesee | 0.50 | 0.36 | 8 |  | NA | NA | NA |
|  | Fairfield | 0.61 | 0.40 | 7 |  | 0.50 | 0.10 | 3 |
|  | Combined | 0.78 | 0.73 | 16 |  | 0.87 | 0.77 | 10 |
| 2019 | Pullman | 0.73 | 0.65 | 5 |  | 0.84 | 0.53 | 5 |
|  | Genesee | 0.65 | 0.43 | 6 |  | NA | NA | NA |
|  | Fairfield ^b^ | 0.47 | 0.37 | 2 |  | NA | NA | NA |
|  | Combined | 0.86 | 0.80 | 20 |  | 0.86 | 0.78 | 7 |

^a^ Only features with ≥ 75% selection occurrence during multiple iterations/runs during model development were considered; ^b^ only data acquired during flowering stage were available for model development; NA indicates failure to develop yield prediction model.

## Supplementary Figures

**Supplementary Figure S1.** Correlation coefficients between image-based features and yield for chickpea yield trial in (a) 2017 and (b) 2018 (plot-by-plot). NDVI: normalized difference vegetation index; GNDVI: green NDVI; SAVI: soil adjusted vegetation index; NDRE: normalized difference red-edge index; and TVI: triangular vegetation index; NDVI, for example, is the average of NDVI values of canopy pixels, while sum NDVI is the sum of NDVI values of canopy pixels; ns: nonsignificant at the 0.05 probability level; significant probability levels: *0.05, **0.01, and ***0.001.


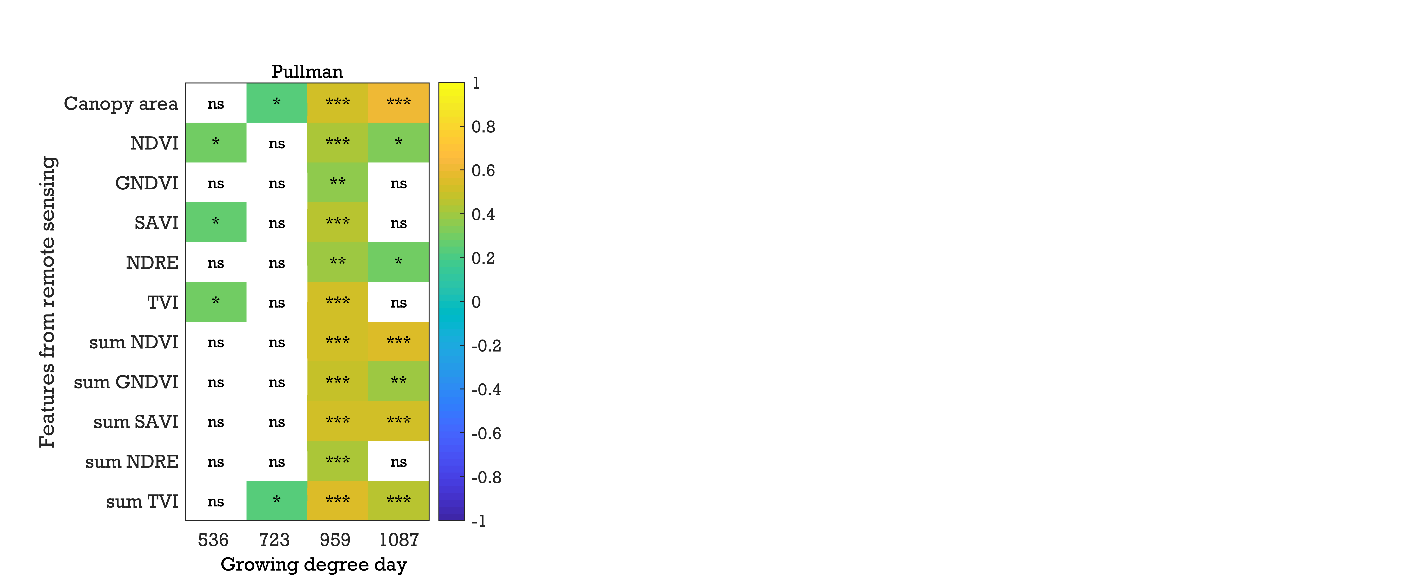

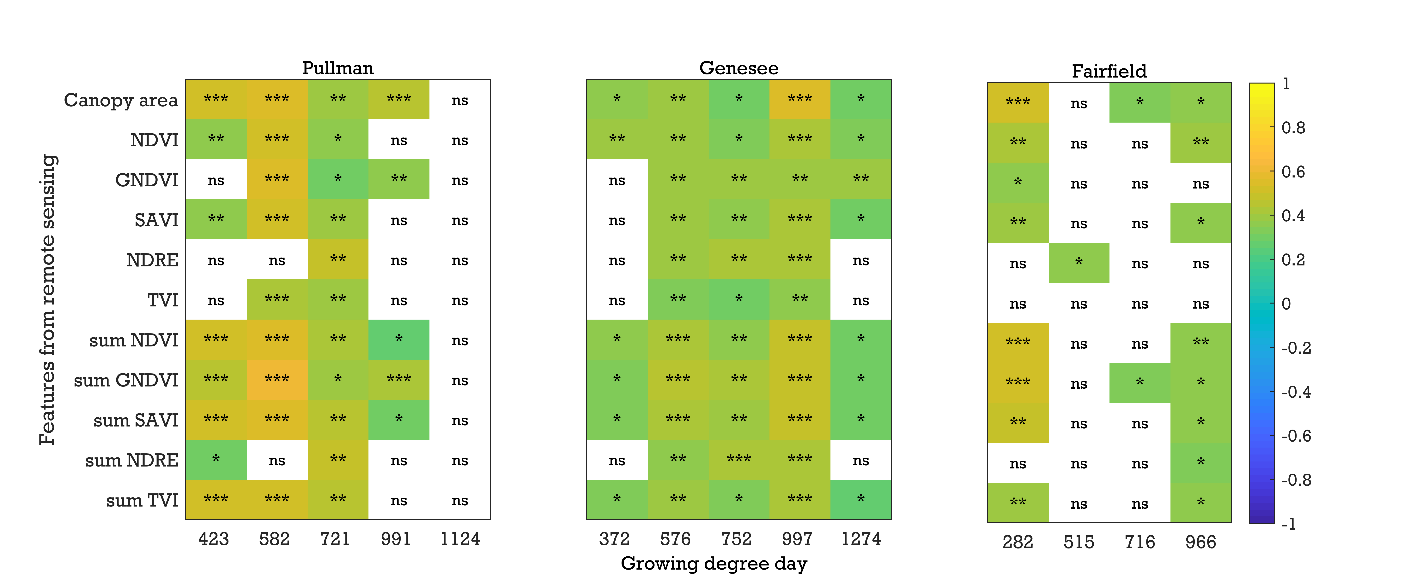


A

B


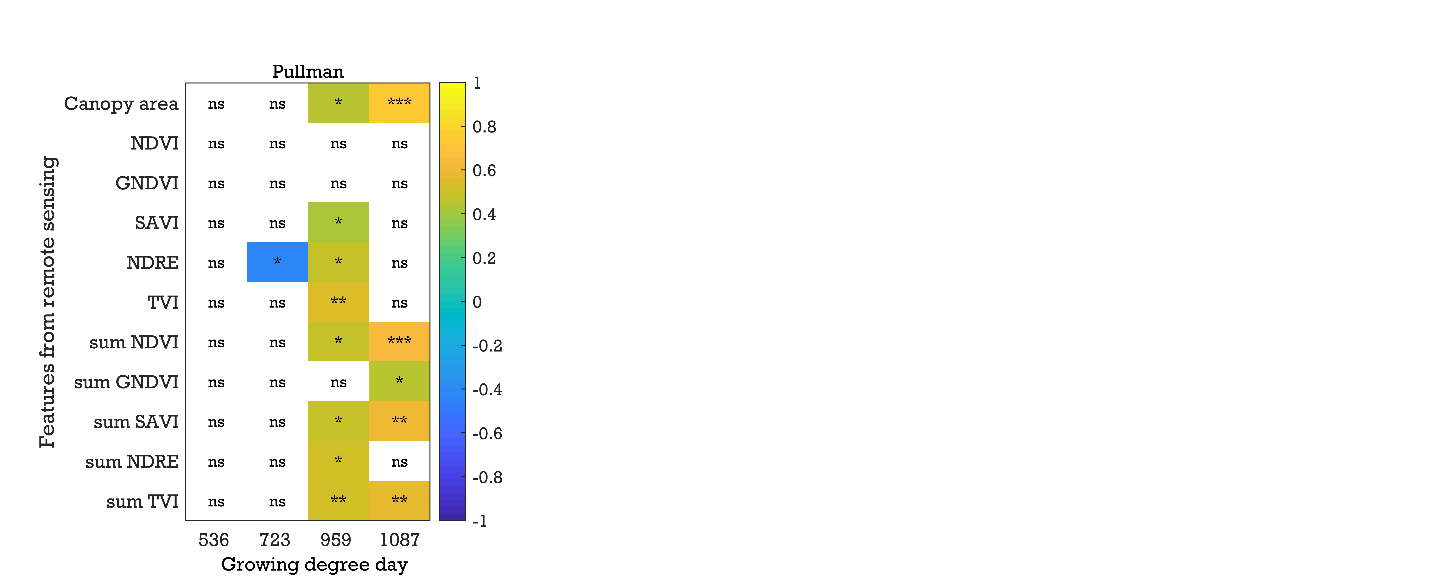

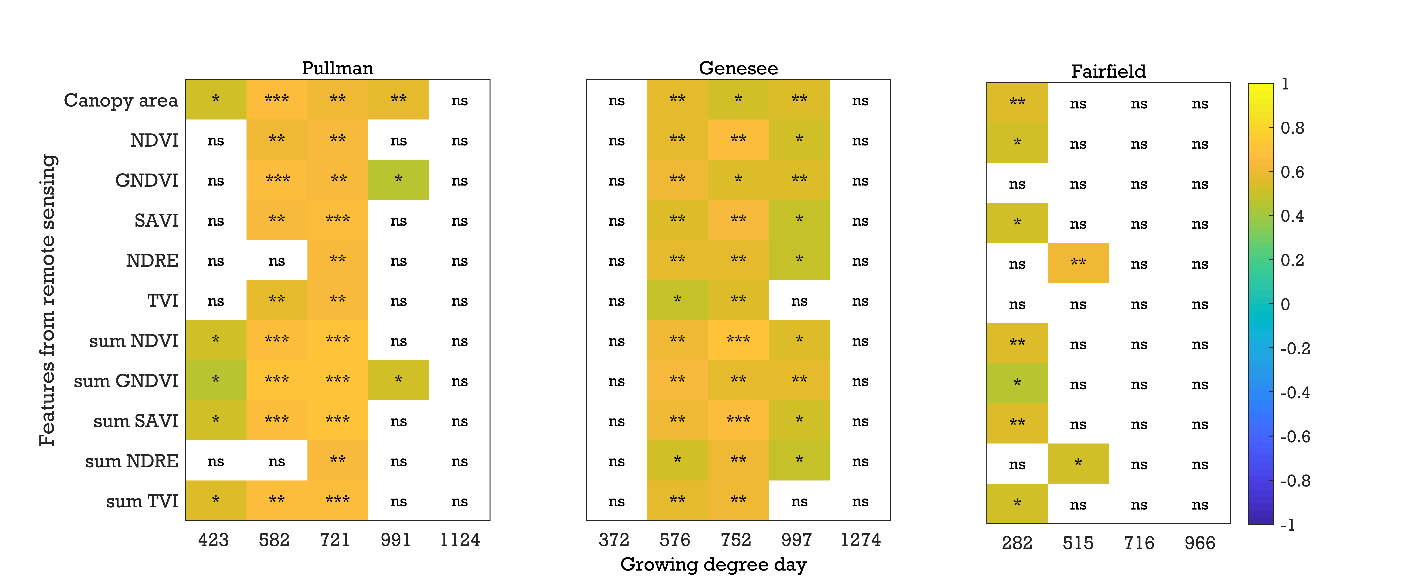


A

B

**Supplementary Figure S2.** Correlation coefficients between image-based features and yield for chickpea yield trial in (a) 2017 and (b) 2018 (cultivar-by-cultivar analysis). NDVI: normalized difference vegetation index; GNDVI: green NDVI; SAVI: soil adjusted vegetation index; NDRE: normalized difference red-edge index; TVI: triangular vegetation index; NDVI, for example, is the average of NDVI values of canopy pixels, while sum NDVI is the sum of NDVI values of canopy pixels. ns: nonsignificant at the 0.05 probability level; significant probability levels: *0.05, **0.01, and ***0.001.

**Supplementary Figure S3**. Correlation coefficients between image-based features and yield for green pea yield trial in (a) 2017 and (b) 2018 (plot-by-plot analysis). NDVI: normalized difference vegetation index; GNDVI: green NDVI; SAVI: soil adjusted vegetation index; NDRE: normalized difference red-edge index; TVI: triangular vegetation index; NDVI, for example, is the average of NDVI values of canopy pixels, while sum NDVI is the sum of NDVI values of canopy pixels. ns: nonsignificant at the 0.05 probability level; significant probability levels: *0.05, **0.01, and ***0.001.


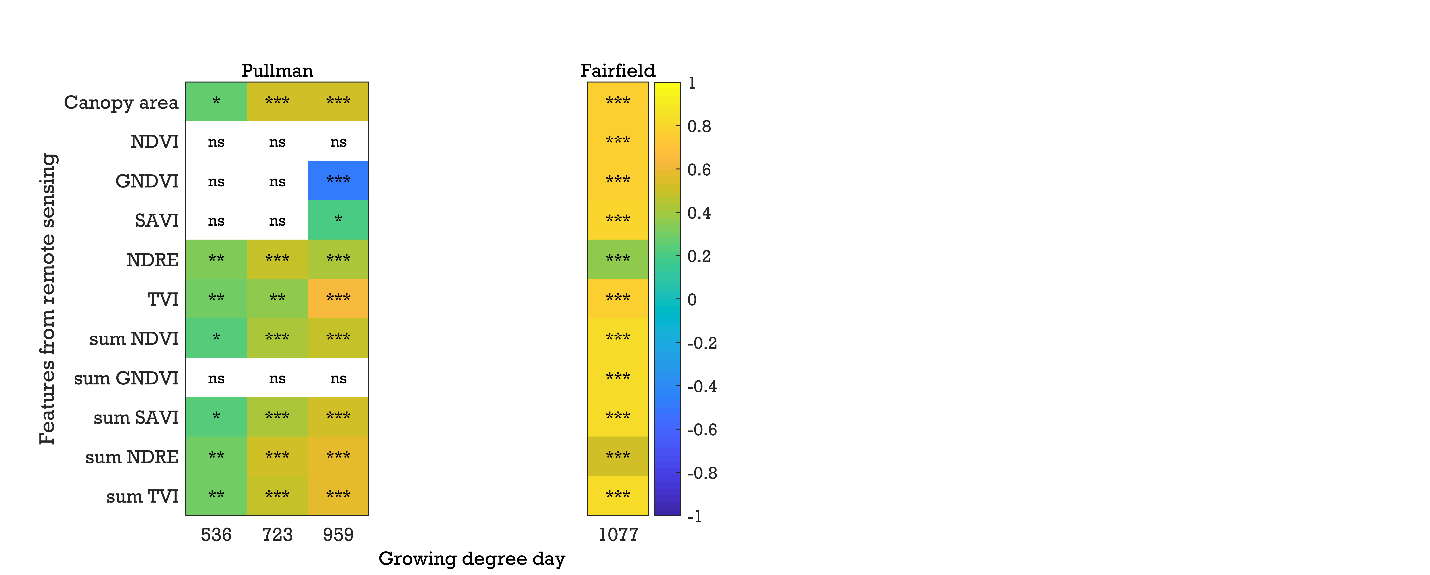

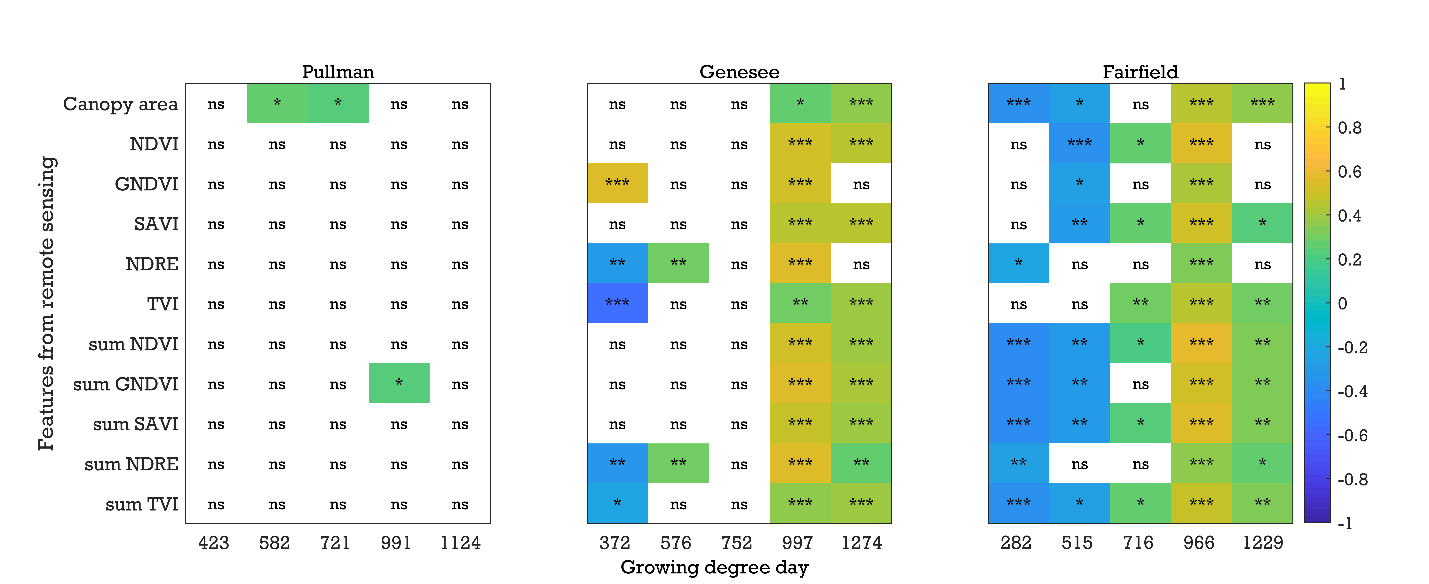


A

B

**Supplementary Figure S4.** Correlation coefficients between image-based features and yield for green pea yield trial in (a) 2017, (b) 2018, and (c) 2019 (cultivar-by-cultivar analysis). NDVI: normalized difference vegetation index; GNDVI: green NDVI; SAVI: soil adjusted vegetation index; NDRE: normalized difference red-edge index; TVI: triangular vegetation index; NDVI, for example, is the average of NDVI values of canopy pixels, while sum NDVI is the sum of NDVI values of canopy pixels. ns: nonsignificant at the 0.05 probability level; significant probability levels: *0.05, **0.01, and ***0.001.


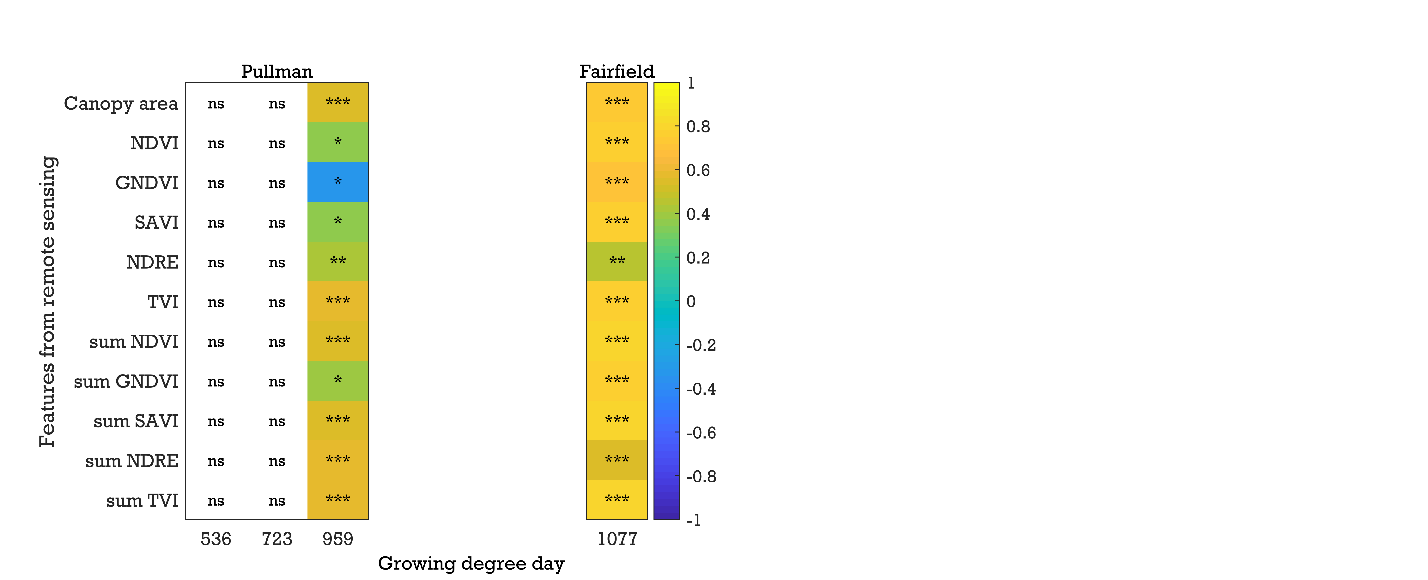

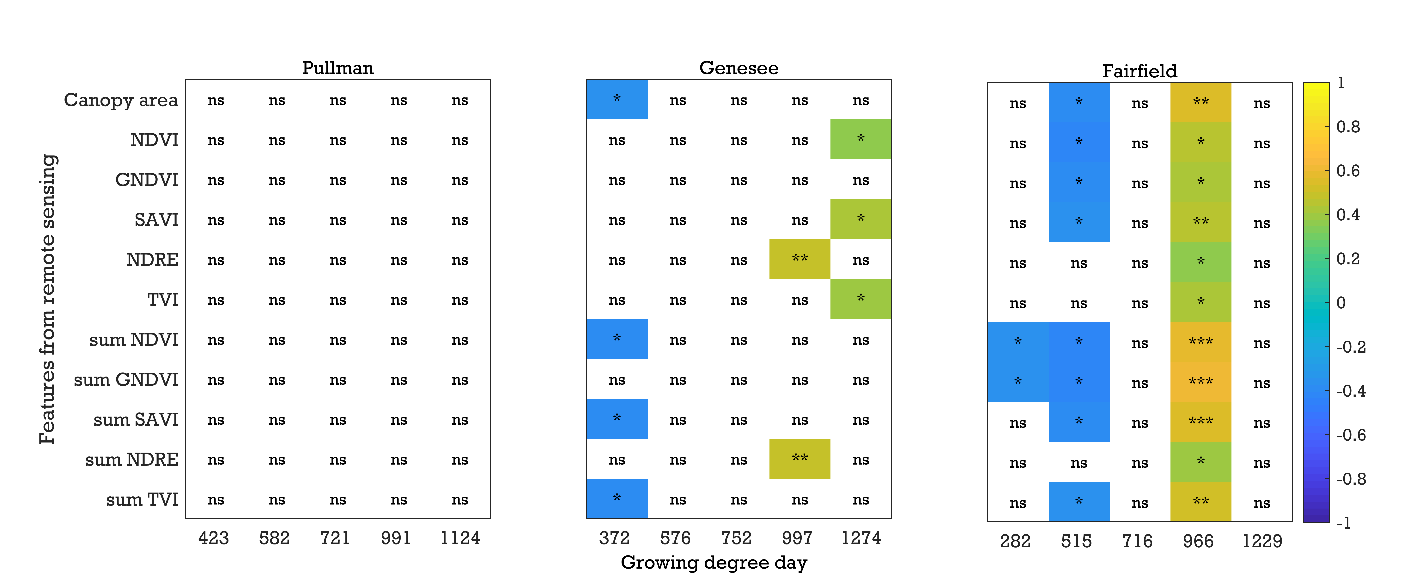

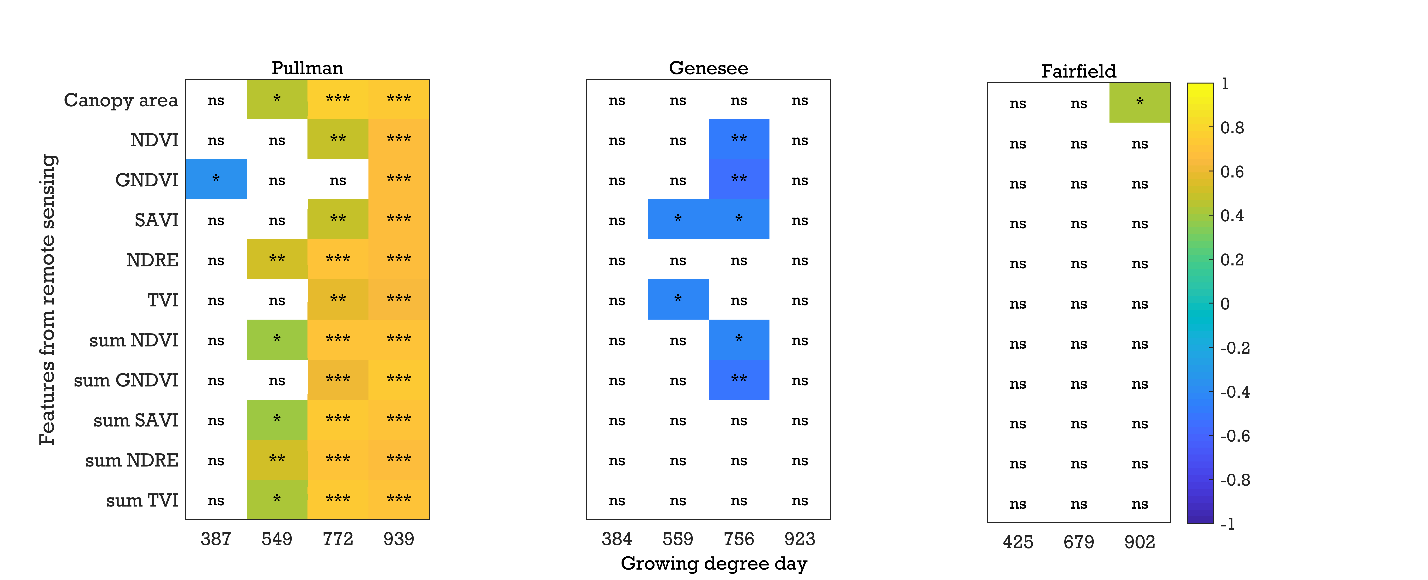


A

B

C

**Supplementary Figure S5.** Correlation coefficients between image-based features and yield for yellow pea yield trial in (a) 2017 and (b) 2018 (plot-by-plot analysis). NDVI: normalized difference vegetation index; GNDVI: green NDVI; SAVI: soil adjusted vegetation index; NDRE: normalized difference red-edge index; TVI: triangular vegetation index; NDVI, for example, is the average of NDVI values of canopy pixels, while sum NDVI is the sum of NDVI values of canopy pixels. ns: nonsignificant at the 0.05 probability level; significant probability levels: *0.05, **0.01, and ***0.001.


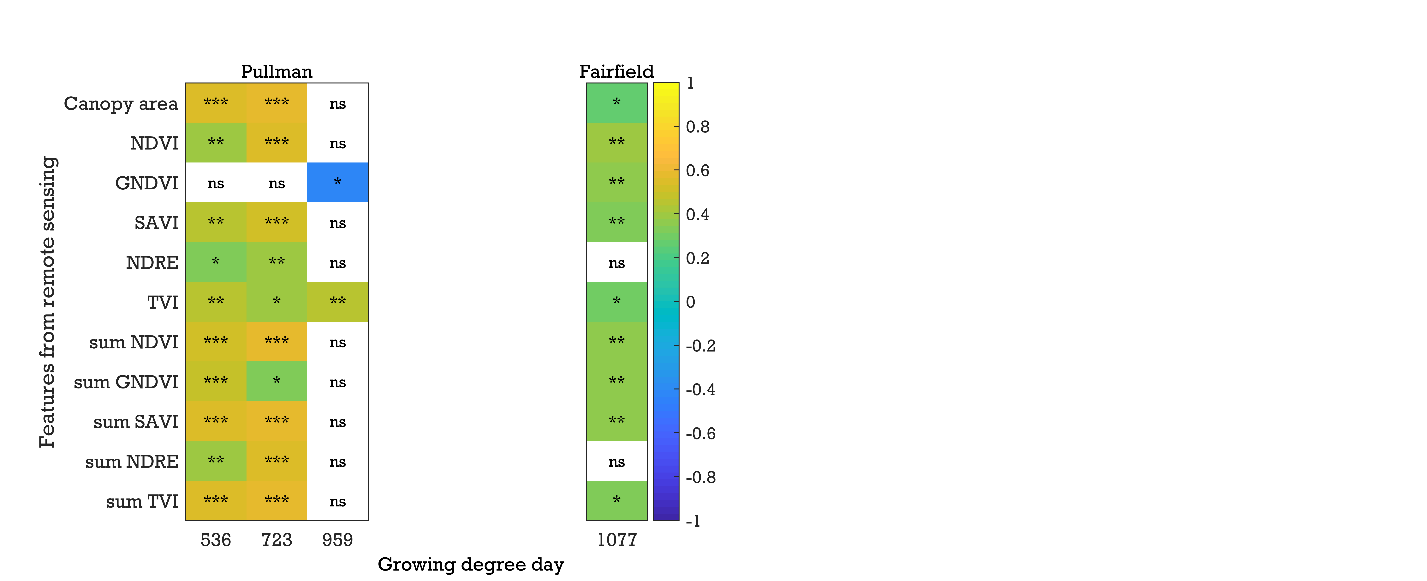

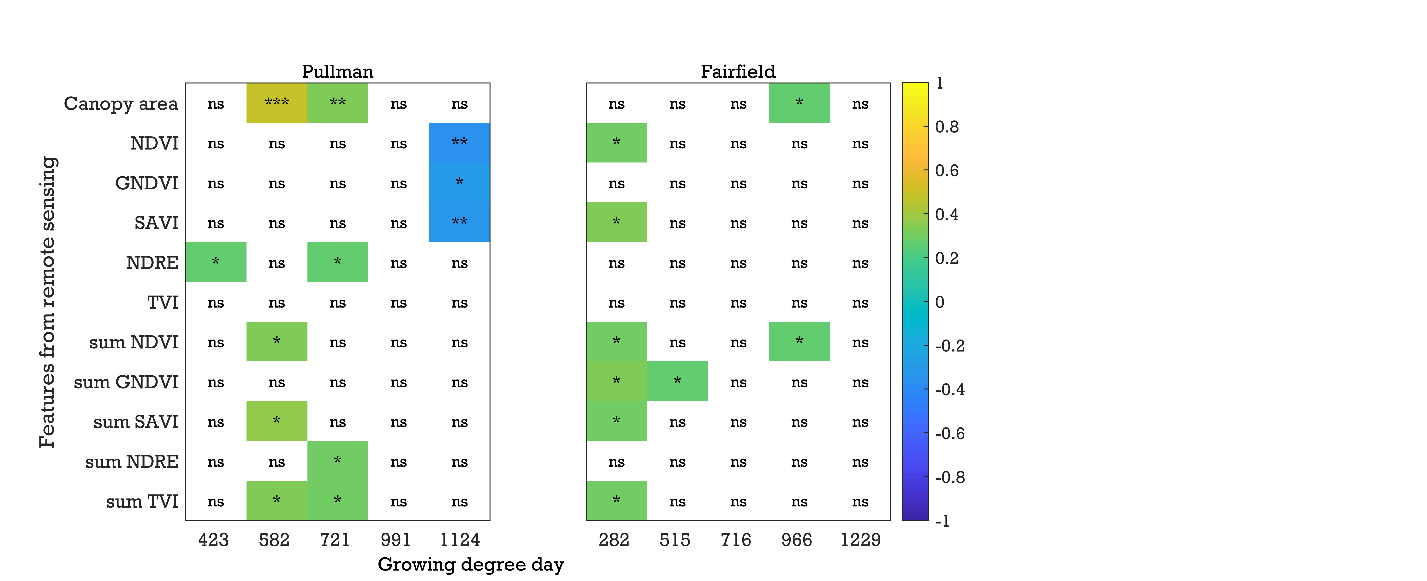


A

B

**Supplementary Figure S6.** Correlation coefficients between image-based features and yield for yellow pea yield trial in (a) 2017, (b) 2018, and (c) 2019 (cultivar-by-cultivar analysis). NDVI: normalized difference vegetation index; GNDVI: green NDVI; SAVI: soil adjusted vegetation index; NDRE: normalized difference red-edge index; TVI: triangular vegetation index; NDVI, for example, is the average of NDVI values of canopy pixels, while sum NDVI is the sum of NDVI values of canopy pixels. ns: nonsignificant at the 0.05 probability level; significant probability levels: *0.05, **0.01, and ***0.001.


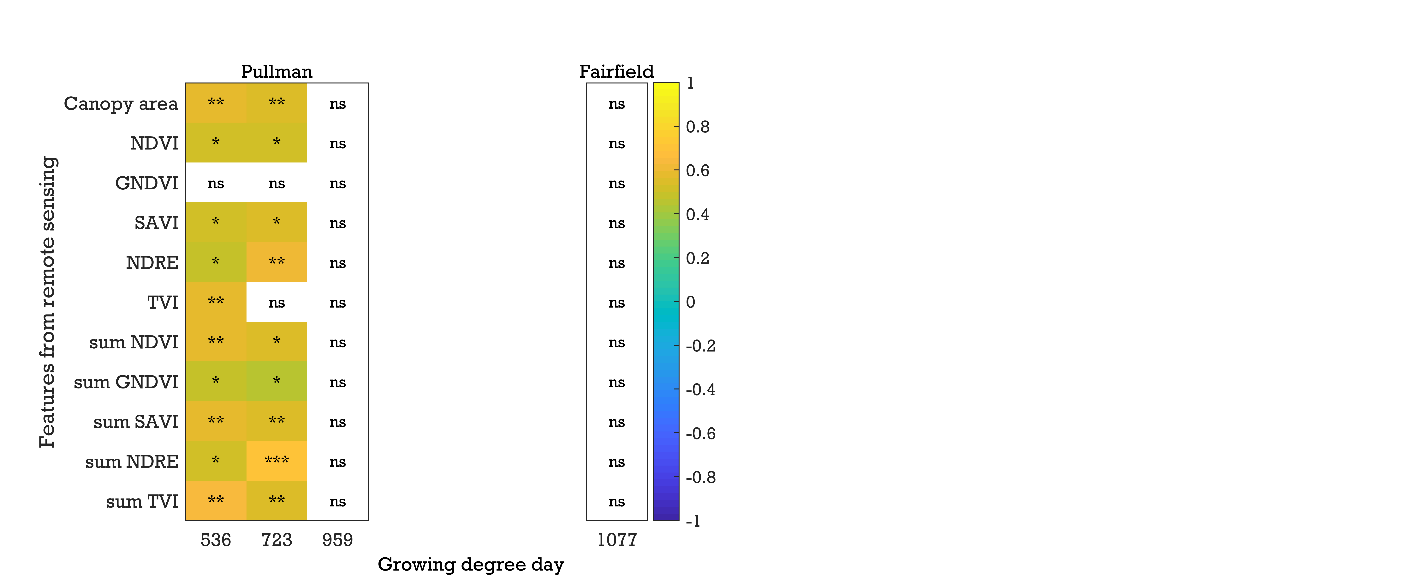

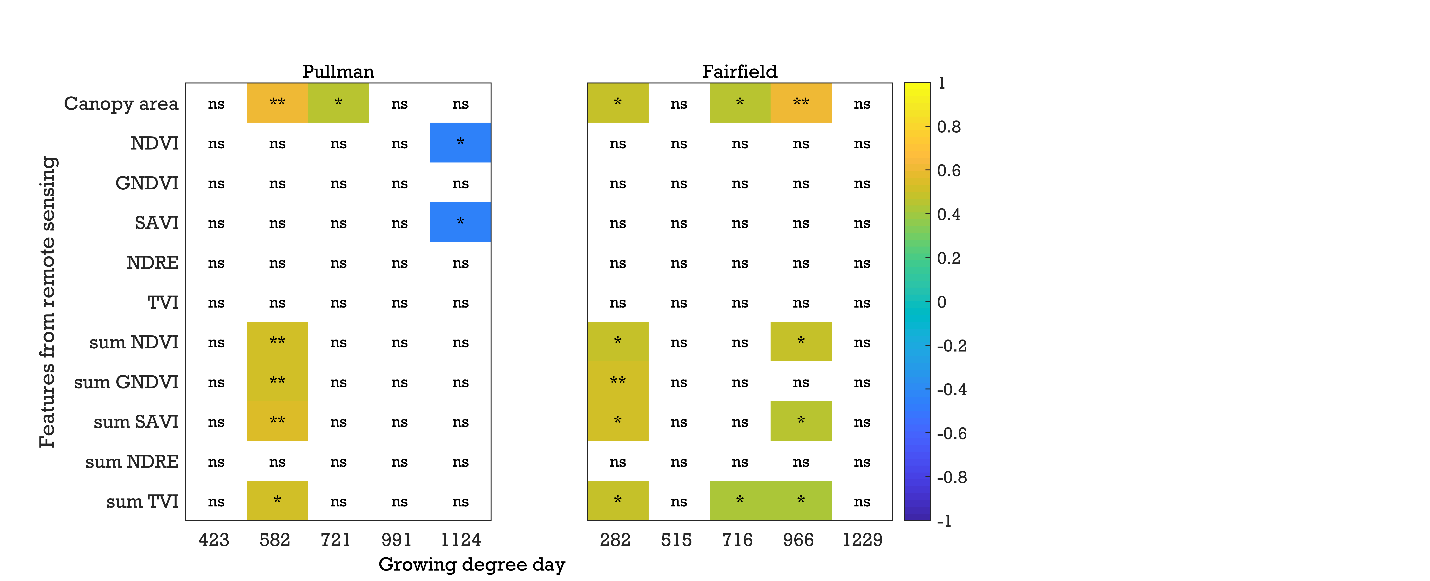

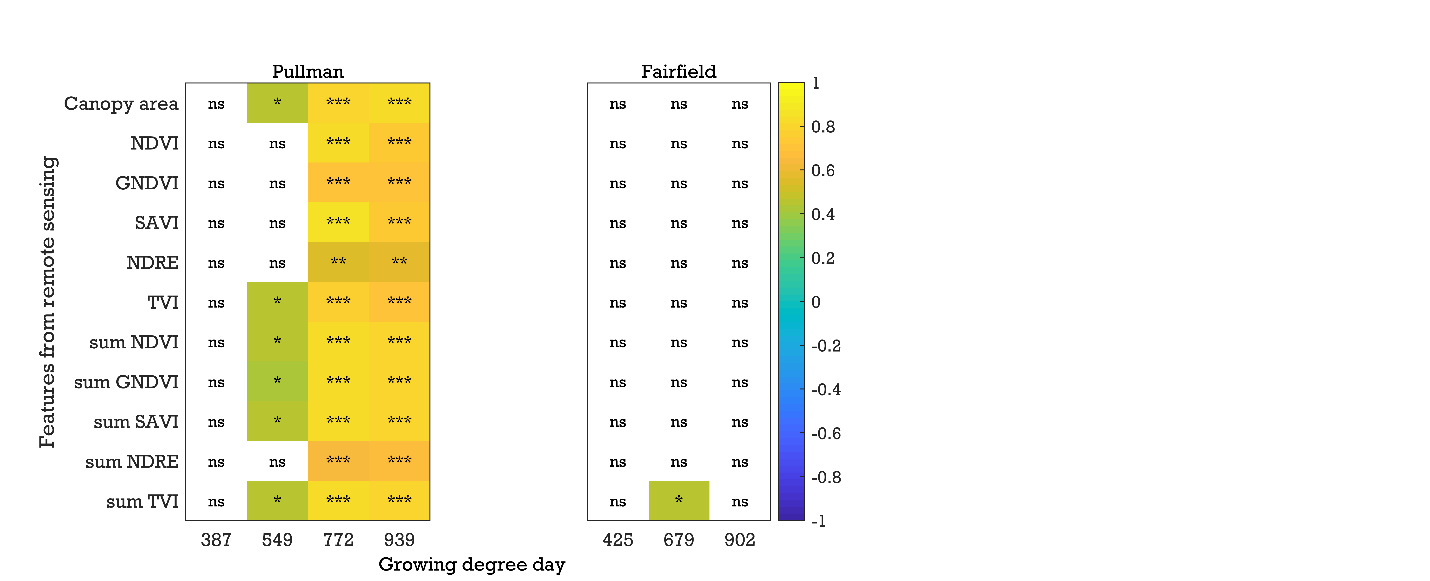


A

B

C

**Supplementary Figure S7.** Correlation coefficients between image-based features and days to 50% flowering for chickpea yield trial: (a) plot-by-plot analysis and (b) cultivar-by-cultivar analysis. NDVI: normalized difference vegetation index; GNDVI: green NDVI; SAVI: soil adjusted vegetation index; NDRE: normalized difference red-edge index; TVI: triangular vegetation index; NDVI, for example, is the average of NDVI values of canopy pixels, while sum NDVI is the sum of NDVI values of canopy pixels; ns: nonsignificant at the 0.05 probability level; significant probability levels: *0.05, **0.01, and ***0.001.


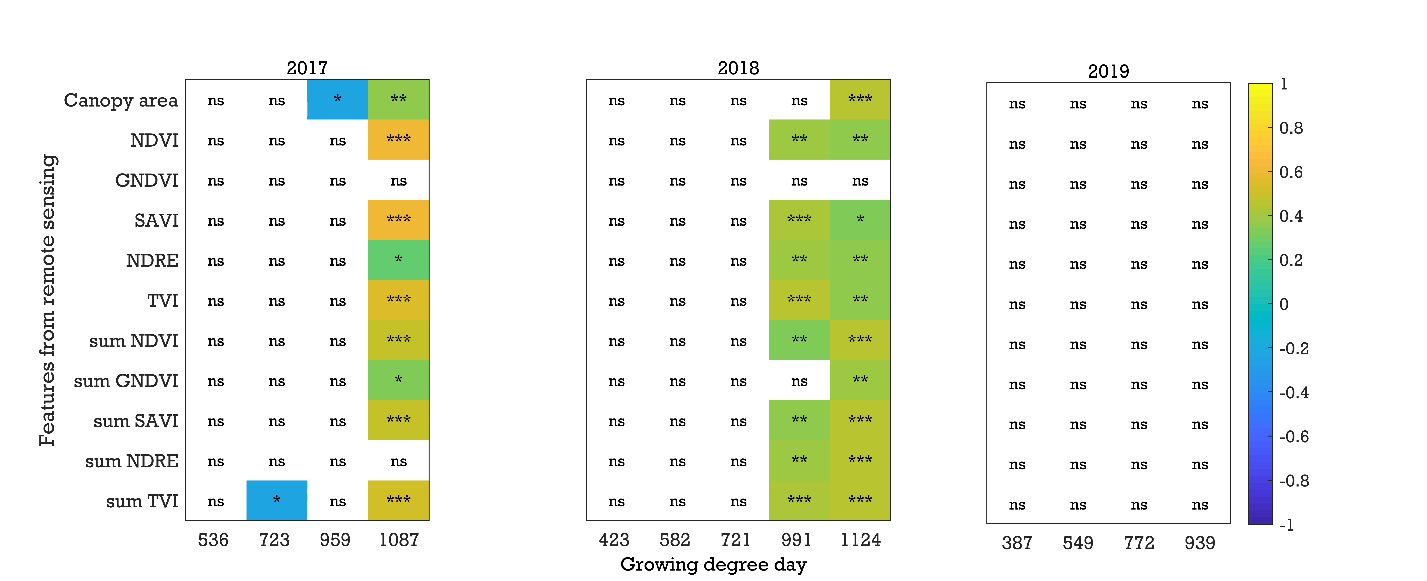

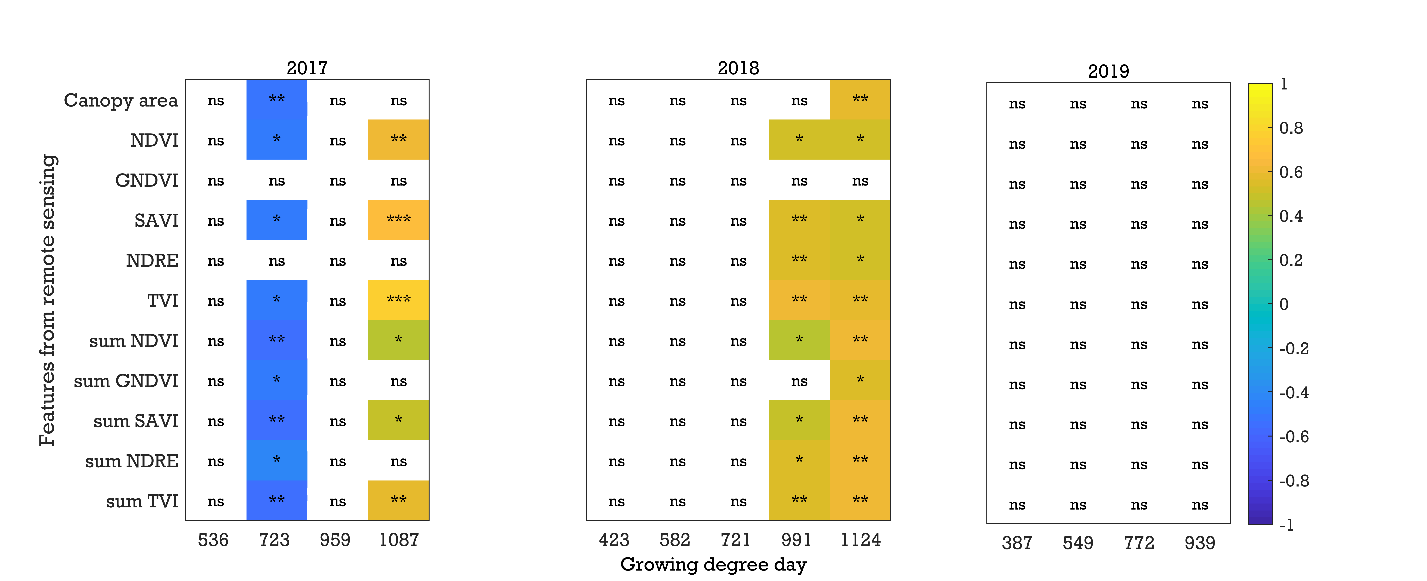


A

B

**Supplementary Figure S8.** Correlation coefficients between image-based features and days to physiological maturity for chickpea yield trial: (a) plot-by-plot analysis and (b) cultivar-by-cultivar analysis. NDVI: normalized difference vegetation index; GNDVI: green NDVI; SAVI: soil adjusted vegetation index; NDRE: normalized difference red-edge index; TVI: triangular vegetation index; NDVI, for example, is the average of NDVI values of canopy pixels, while sum NDVI is the sum of NDVI values of canopy pixels, ns: nonsignificant at the 0.05 probability level; significant probability levels: *0.05, **0.01, and ***0.001.


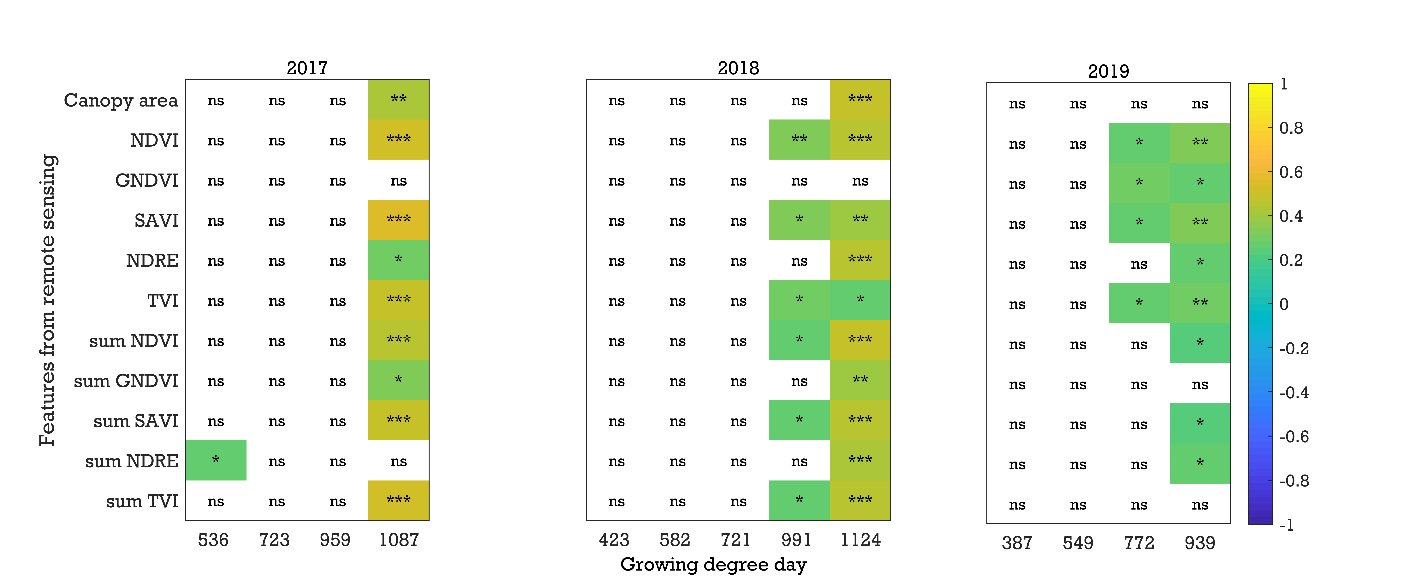

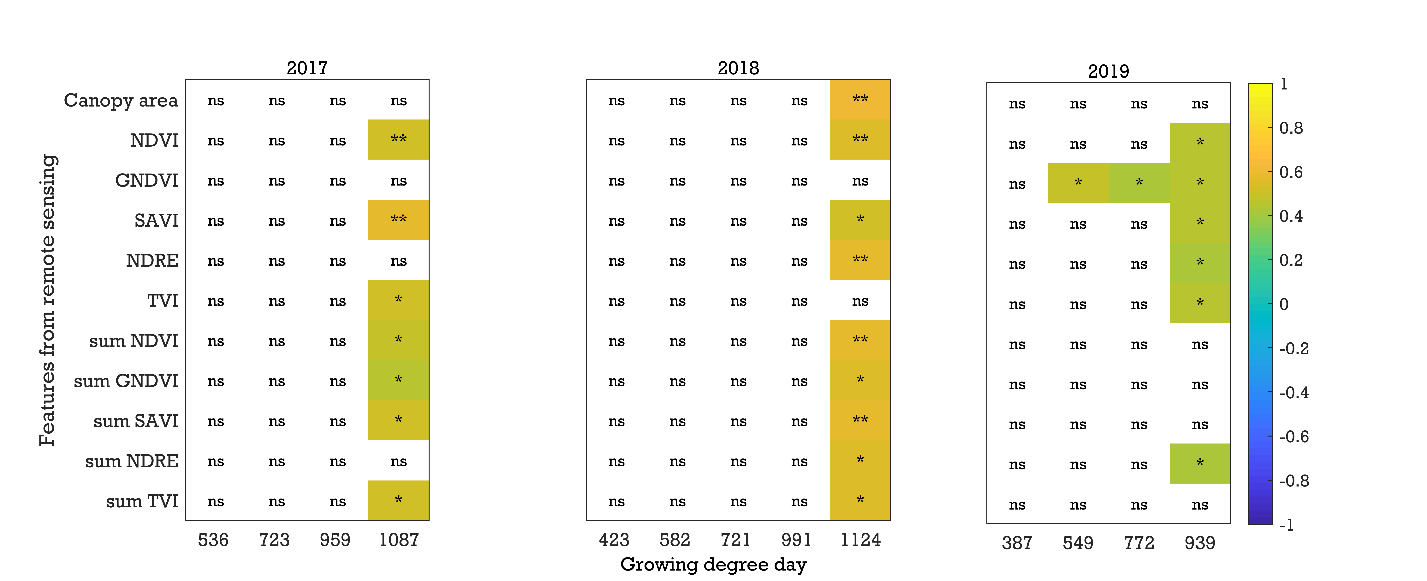


A

B

**Supplementary Figure S9.** Correlation coefficients between image-based features and days to 50% flowering for green pea yield trial: (a) plot-by-plot analysis and (b) cultivar-by-cultivar analysis. NDVI: normalized difference vegetation index; GNDVI: green NDVI; SAVI: soil adjusted vegetation index; NDRE: normalized difference red-edge index; TVI: triangular vegetation index; NDVI, for example, is the average of NDVI values of canopy pixels, while sum NDVI is the sum of NDVI values of canopy pixels; ns: nonsignificant at the 0.05 probability level; significant probability levels: *0.05, **0.01, and ***0.001.


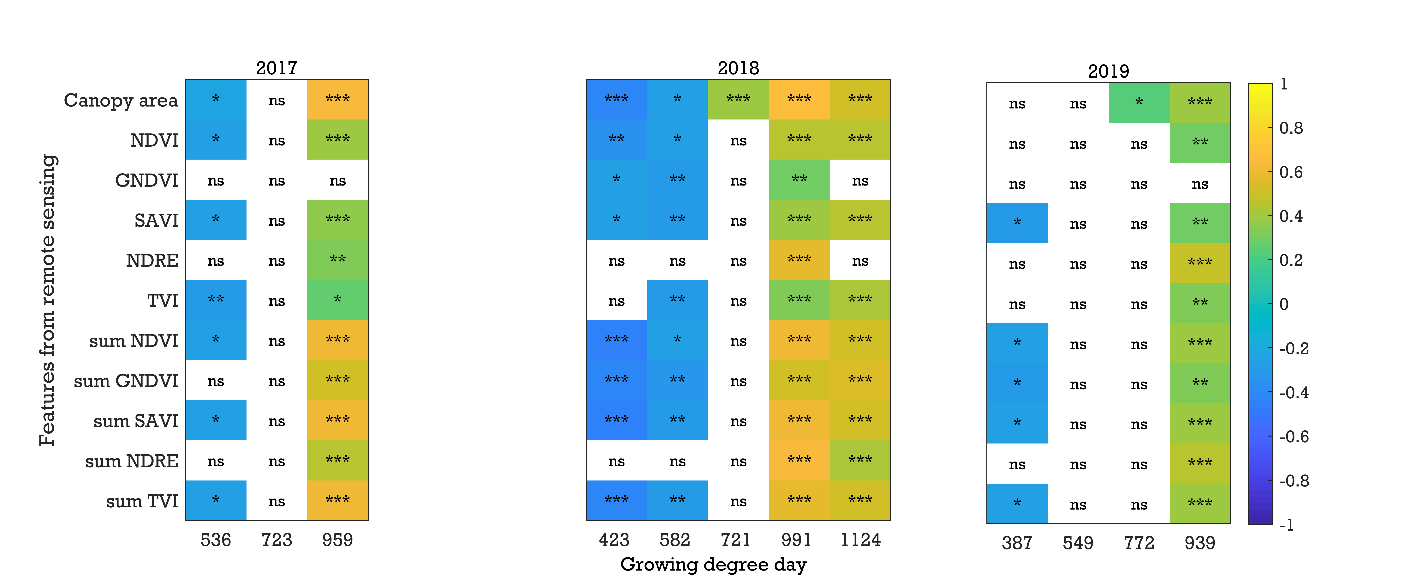

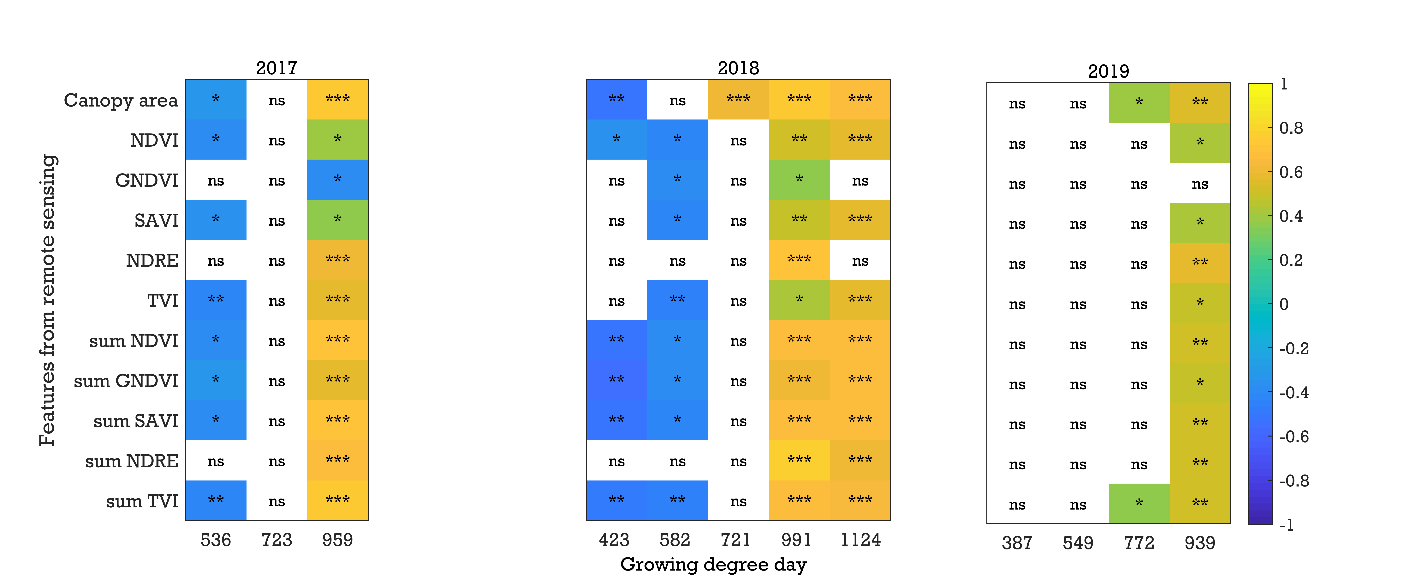


A

B

**Supplementary Figure S10.** Correlation coefficients between image-based features and days to physiological maturity for green pea yield trial: (a) plot-by-plot analysis and (b) cultivar-by-cultivar analysis. NDVI: normalized difference vegetation index; GNDVI: green NDVI; SAVI: soil adjusted vegetation index; NDRE: normalized difference red-edge index; TVI: triangular vegetation index; NDVI, for example, is the average of NDVI values of canopy pixels, while sum NDVI is the sum of NDVI values of canopy pixels; ns: nonsignificant at the 0.05 probability level; significant probability levels: *0.05, **0.01, and ***0.001.


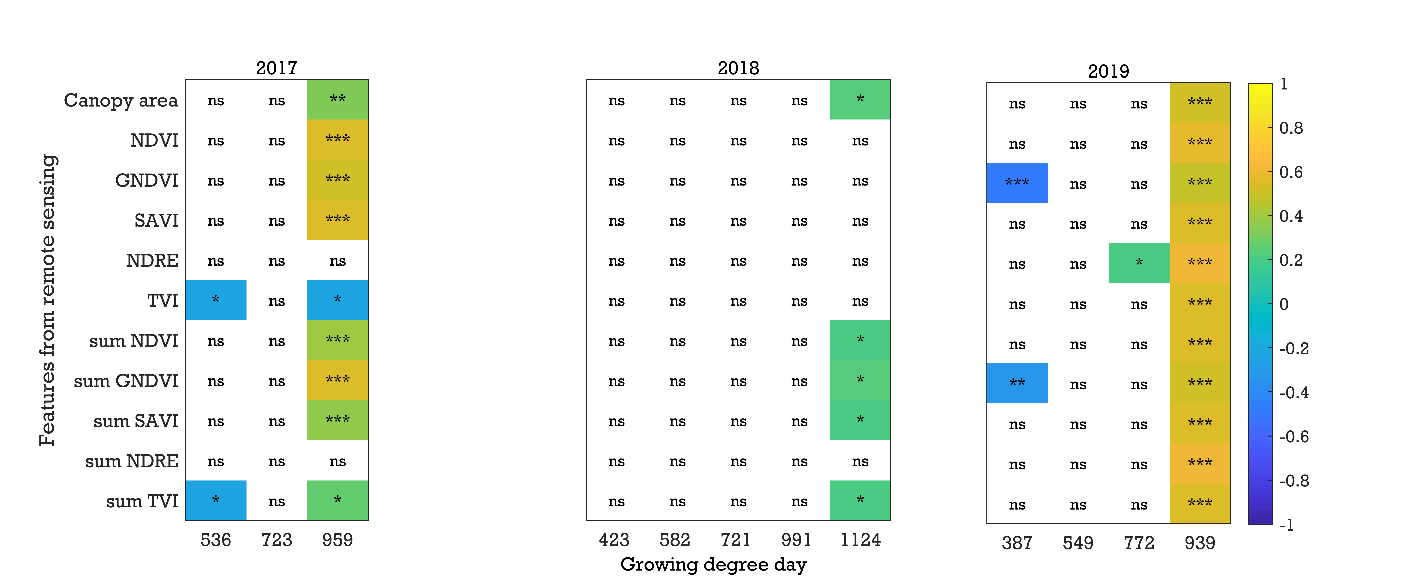

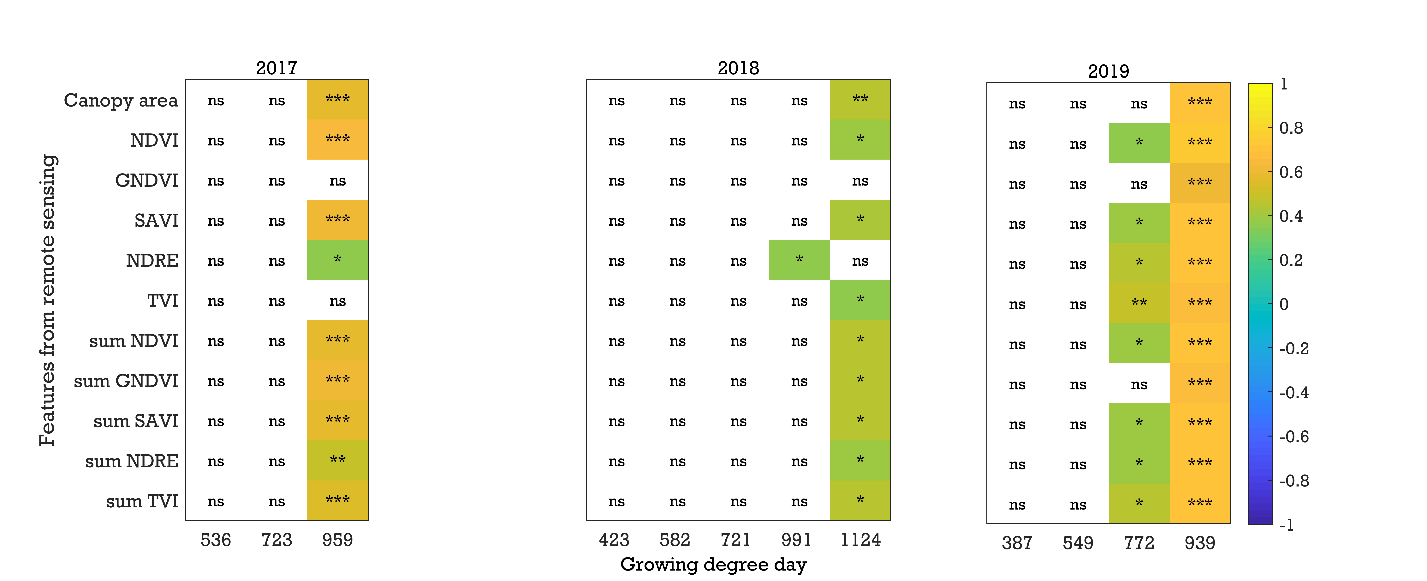


A

B

**Supplementary Figure S11.** Correlation coefficients between image-based features and days to 50% flowering for yellow pea yield trial: (a) plot-by-plot analysis and (b) cultivar-by-cultivar. NDVI: normalized difference vegetation index; GNDVI: green NDVI; SAVI: soil adjusted vegetation index; NDRE: normalized difference red-edge index; TVI: triangular vegetation index; NDVI, for example, is the average of NDVI values of canopy pixels, while sum NDVI is the sum of NDVI values of canopy pixels; ns: nonsignificant at the 0.05 probability level; significant probability levels: *0.05, **0.01, and ***0.001.


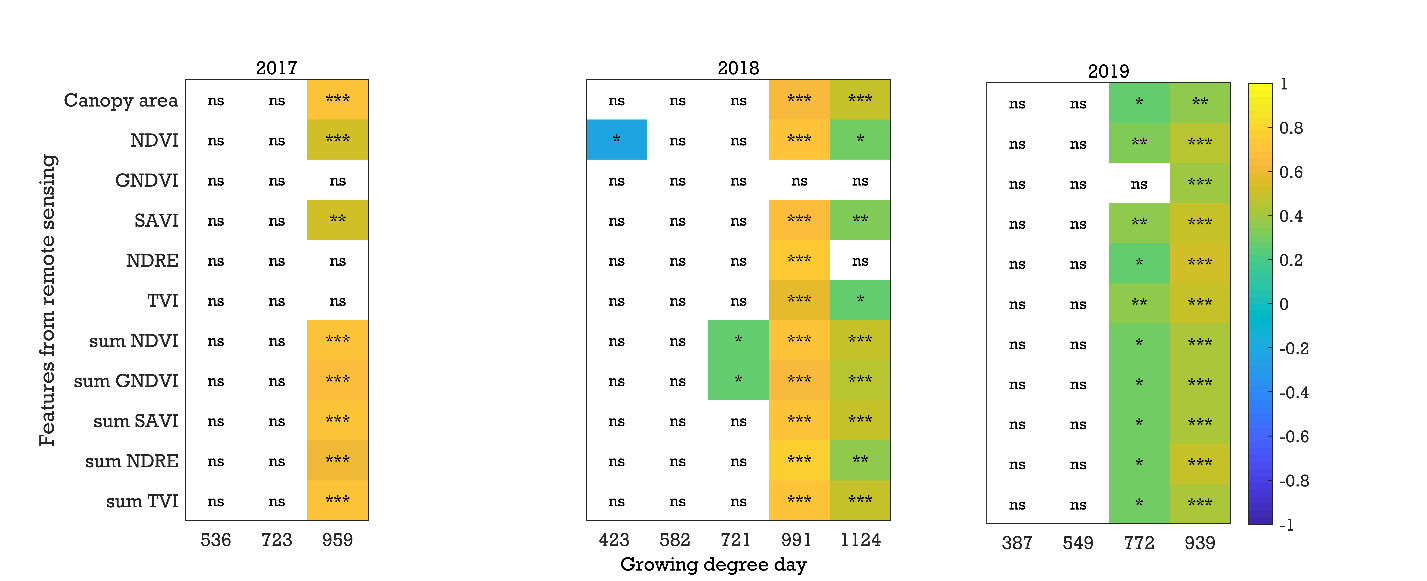

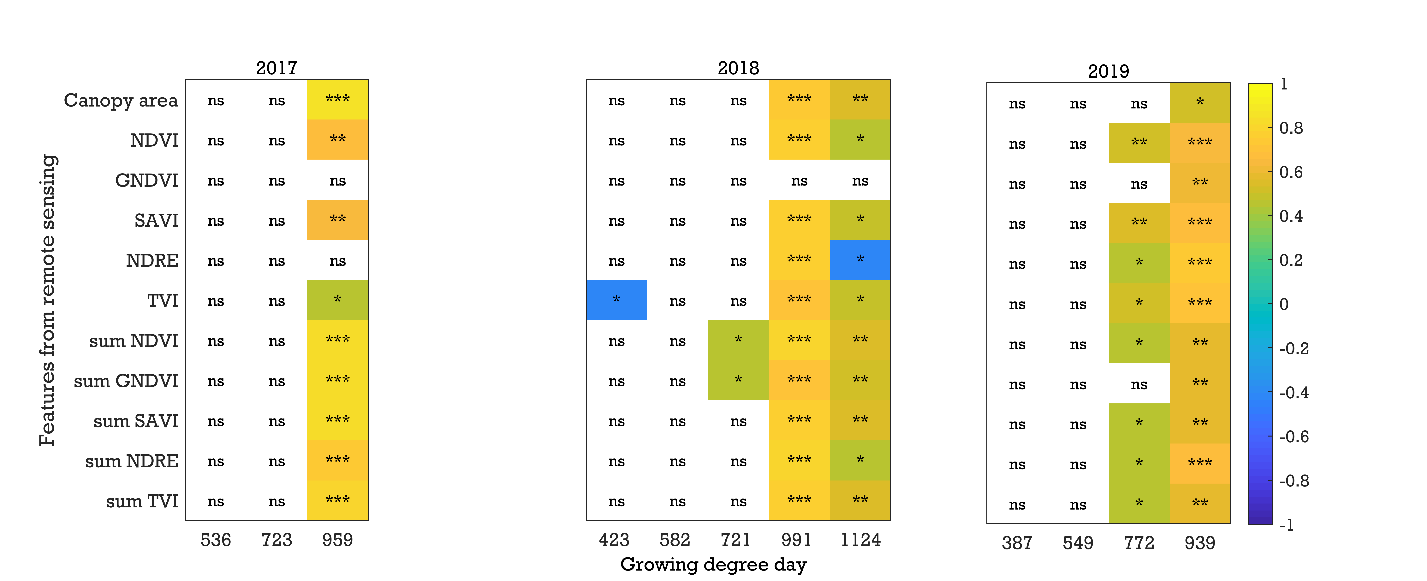


A

B

**Supplementary Figure S12.** Correlation coefficients between image-based features and days to physiological maturity for yellow pea yield trial: (a) plot-by-plot analysis and (b) cultivar-by-cultivar analysis. NDVI: normalized difference vegetation index; GNDVI: green NDVI; SAVI: soil adjusted vegetation index; NDRE: normalized difference red-edge index; TVI: triangular vegetation index; NDVI, for example, is the average of NDVI values of canopy pixels, while sum NDVI is the sum of NDVI values of canopy pixels; ns: nonsignificant at the 0.05 probability level; significant probability levels: *0.05, **0.01, and ***0.001.


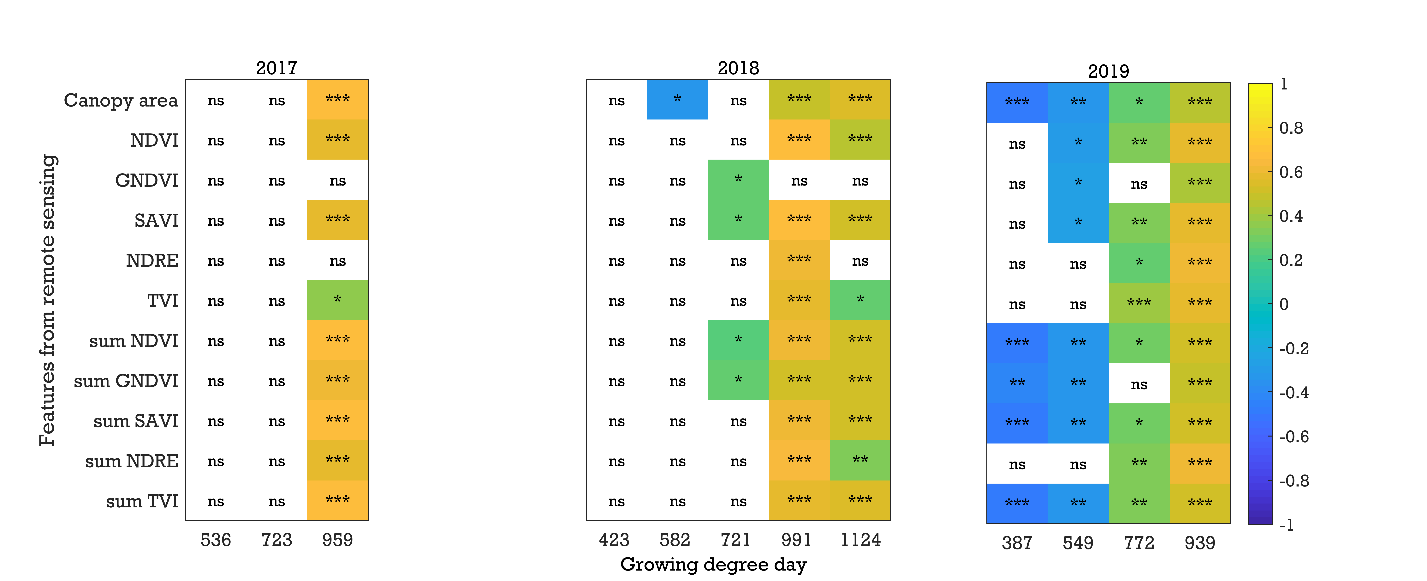

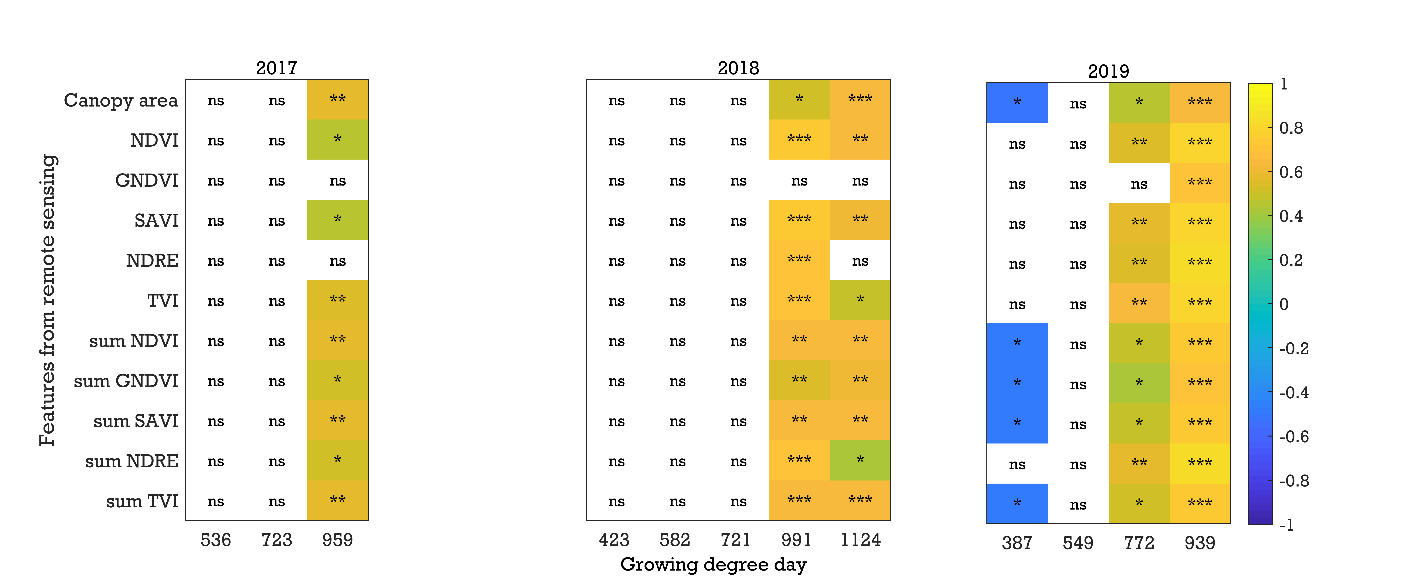


A

B
